# Brain–body mitochondrial distribution patterns lack coherence and point to tissue-specific regulatory mechanisms

**DOI:** 10.1093/lifemeta/loaf012

**Published:** 2025-04-12

**Authors:** Jack Devine, Anna S Monzel, David Shire, Ayelet M Rosenberg, Alex Junker, Alan A Cohen, Martin Picard

**Affiliations:** Division of Behavioral Medicine, Department of Psychiatry, Columbia University Irving Medical Center, New York, NY 10032, United States; Division of Behavioral Medicine, Department of Psychiatry, Columbia University Irving Medical Center, New York, NY 10032, United States; Division of Behavioral Medicine, Department of Psychiatry, Columbia University Irving Medical Center, New York, NY 10032, United States; Division of Behavioral Medicine, Department of Psychiatry, Columbia University Irving Medical Center, New York, NY 10032, United States; Division of Behavioral Medicine, Department of Psychiatry, Columbia University Irving Medical Center, New York, NY 10032, United States; Robert N Butler Columbia Aging Center, Columbia University Mailman School of Public Health, New York, NY 10032, United States; Department of Environmental Health Sciences, Columbia University Mailman School of Public Health, New York, NY 10032, United States; Division of Behavioral Medicine, Department of Psychiatry, Columbia University Irving Medical Center, New York, NY 10032, United States; Robert N Butler Columbia Aging Center, Columbia University Mailman School of Public Health, New York, NY 10032, United States; New York State Psychiatric Institute, New York, NY 10032, United States; Department of Neurology, H. Houston Merritt Center, Columbia Translational Neuroscience Initiative, Columbia University Irving Medical Center, New York, NY 10032, United States

**Keywords:** mitochondrion, gene regulation, mitochondrial biogenesis, energy sensing, inter-organ crosstalk, disease risk

## Abstract

Energy transformation capacity is generally assumed to be a coherent individual trait driven by genetic and environmental factors. This predicts that some individuals should have consistently high, while others show consistently low mitochondrial oxidative phosphorylation (OxPhos) capacity across organ systems. Here, we test this assumption using multi-tissue molecular and enzymatic assays in mice and humans. Across up to 22 mouse tissues, neither mitochondrial OxPhos capacity nor mitochondrial DNA (mtDNA) density was correlated between tissues (median *r* = −0.01 to 0.16), indicating that animals with high mitochondrial content or capacity in one tissue may have low content or capacity in other tissues. Similarly, RNA sequencing (RNAseq)-based indices of mitochondrial expression across 45 tissues from 948 women and men (genotype-tissue expression [GTEx]) showed only small to moderate coherence between some tissues, such as between brain regions (*r* = 0.26), but not between brain–body tissue pairs (*r* = 0.01). The mtDNA copy number (mtDNAcn) also lacked coherence across human tissues. Mechanistically, tissue-specific differences in mitochondrial gene expression were partially attributable to (i) tissue-specific activation of energy sensing pathways, including the transcriptional coactivator peroxisome proliferator-activated receptor gamma coactivator 1-alpha (PGC-1α), the integrated stress response (ISR), and other molecular regulators of mitochondrial biology, and (ii) proliferative activity across tissues. Finally, we identify subgroups of individuals with distinct mitochondrial distribution strategies that map onto distinct clinical phenotypes. These data raise the possibility that tissue-specific energy sensing pathways may contribute to idiosyncratic mitochondrial distribution patterns among individuals.

## Introduction

**Figure 1:**
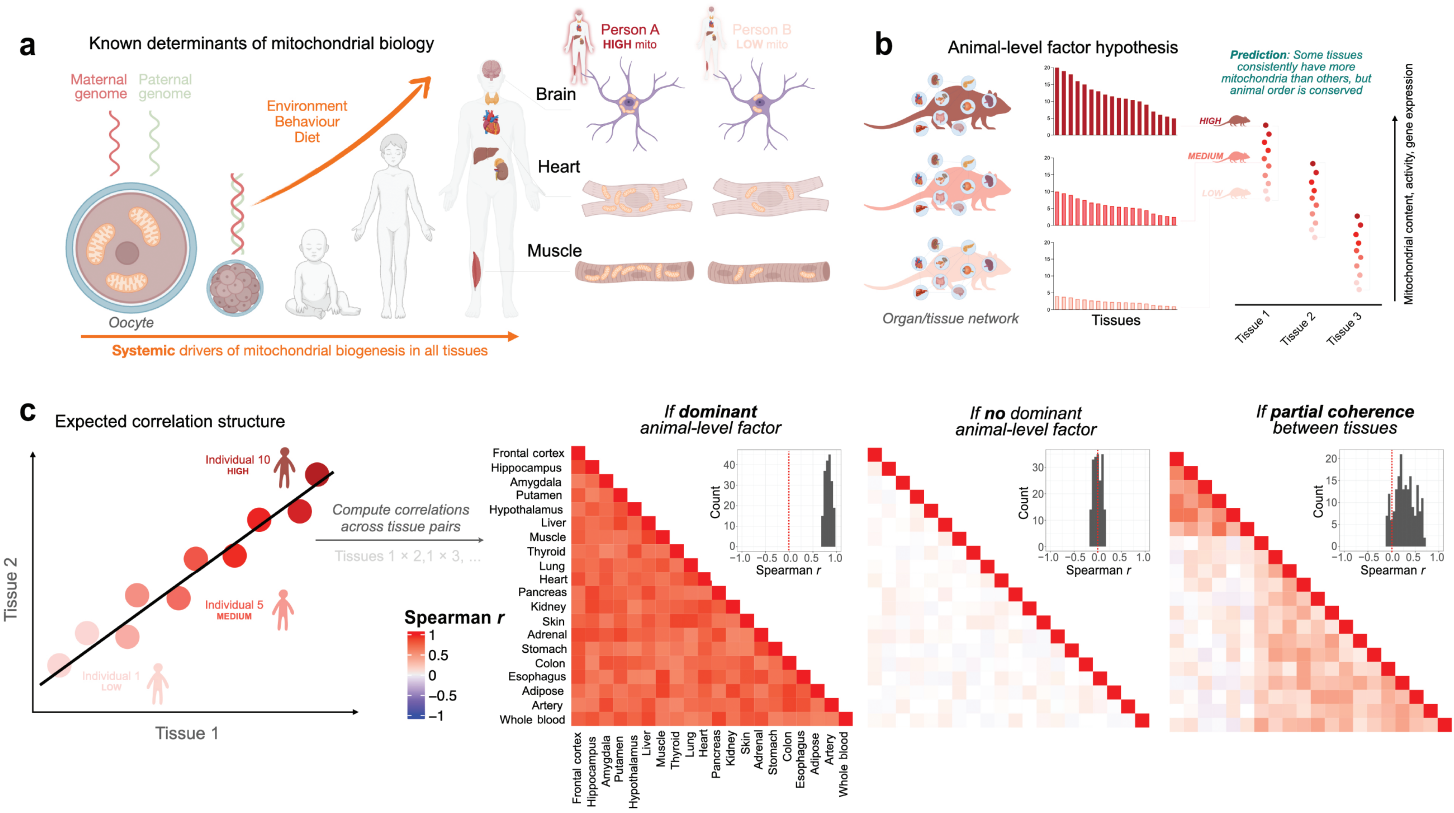
Expectations and hypotheses on individual phenotypes of multi-tissue mitochondrial biology. Figure illustrating the multi-tissue coherence hypothesis. The expectation is that some individuals will have high mitochondrial content in all tissues and some will have low mitochondrial content in all tissues. This would result in strong correlations between tissues in measures of mitochondrial biology.

A major driver of organ-specific function and dysfunction is the capacity for energy transformation. Energy enables organ-specific function and inter-organ communication [1, 2]. In breathing animals, energy is transformed within mitochondria, where the oxidative phosphorylation (OxPhos) system converts oxygen and food substrates into usable cellular energy. Because we develop from a single fertilized mitochondria-filled oocyte into a mature adult composed of genetically identical cells and mitochondria, it is generally assumed that inherited (epi)genetic factors define mitochondrial OxPhos capacity and biology homogenously across the whole body. In single-tissue studies such as blood immune cells, genetic variants explain a portion of the variance in mitochondrial DNA (mtDNA) copy number (mtDNAcn) [3]. Similarly, exercise can induce biogenesis not only in the working muscles but also in the brain and other tissues [4, 5], suggesting that behaviorally driven mitochondrial adaptations occur systemically across the whole body, via the action of only partially resolved factors [6]. Therefore, based on these premises, there is an expectation that organisms should display mitochondrial inter-tissue “coherence”. This means that relative to other individuals in the population, an individual who has high mitochondrial content in one tissue (e.g. brain) would also have high mitochondrial content in other tissues (e.g. heart, skeletal muscle, skin, etc.). Similarly, we would expect some individuals to have low mitochondrial content across all tissues (Fig. 1a).

If the inter-tissue coherence hypothesis is true, measurements of mitochondrial OxPhos capacity and gene expression across multiple tissues would be expected to resemble Fig. 1b. Tissues with high baseline energetic demand (e.g. heart) consistently have more mitochondria than others with lower demand. Most importantly, the animal order should be conserved across tissues: some animals should exhibit high values in all tissues, and some animals should be low in all tissues. The inter-tissue correlation structure observed from this result would be strongly positive, reflecting coherence between tissues (Fig. 1c). However, if a different correlation structure is observed between tissues (weak, absent, or negative correlations), this would suggest that the individual-level, inter-tissue coherence hypothesis is incorrect.

The assumption of inter-tissue coherence underlies much biological research, although rarely made explicit and seldom tested, with some exceptions. In non-human primates, the maximal mitochondrial respiratory capacity of both monocytes and platelets was correlated with permeabilized skeletal muscle maximal respiratory capacity and isolated cardiac muscle respiratory control ratio [7]. Also, in non-human primates, the maximal respiration of CD14^+^ monocytes and frontal cortex were found to be significantly correlated [8]. One study in humans also found platelet maximal respiration to be correlated with muscle maximal respiration [9]. These results largely derived from small studies suggest that mitochondrial respiratory capacity is an individual-level systemic trait. However, several other human studies have not observed a positive correlation between blood cells and skeletal muscle in mitochondrial respiration [10–12], and several more negative studies may be unreported. In particular, a robust study comparing mtDNAcn across 12 human tissues in 152 individuals found no correlation between most tissues [13]. This lack of coherence in mitochondrial biology across human tissues, together with recent evidence that different human organs age at different rates relative to one another, within a given organism [14–16], brings the inter-tissue mitochondrial coherence hypothesis into question.

Here, we systematically test at scale this inter-tissue coherence hypothesis using multi-tissue OxPhos enzymatic activity measurements in two independent mouse cohorts, plus in whole transcriptomes from 45 tissues from 948 women and men (*n* = 16,205 samples, *n* = 983 total tissue-tissue pairs). Unlike the default hypothesis that mitochondrial biology is predominantly an organism-level attribute of each individual, our results highlight the general lack of coherence across brain and non-brain organ systems. These results point towards the emergence of idiosyncratic mitochondrial distribution patterns across individuals, potentially driven by genetic regulatory pathways associated with organ-specific mitochondrial gene expression patterns.

## Results

### Multi-tissue mitochondrial enzymatic activities and mtDNA density display low between-tissue correlations in two independent mouse cohorts

We first tested the trait-level inter-tissue coherence hypothesis using direct biochemical enzymatic activities for OxPhos complexes I, II, and IV, and the Krebs cycle enzyme citrate synthase (CS) in five tissues: brain—hippocampus; non-brain—liver, brown fat, muscle, and bone from 16 male mice. The mtDNA density was also quantified in each tissue by quantitative PCR (qPCR) (*n* = 10 tissue pairs × 5 measures per tissue, giving a total of *n* = 50 pairwise comparisons for specific mitochondrial features compared between tissues). Contrary to the hypothesis, the resulting correlation matrix of the five mitochondrial measures across the five tissues showed minimal or even negative correlation between tissues (Supplementary Fig. S1). The frequency distribution of the correlation coefficients (Spearman *r*) across the 50 tissue pairs is shown in Supplementary Fig. S1b. Individual biplots in which each data point represents a different animal show small-to-null correlations across organ systems.

**Figure 2:**
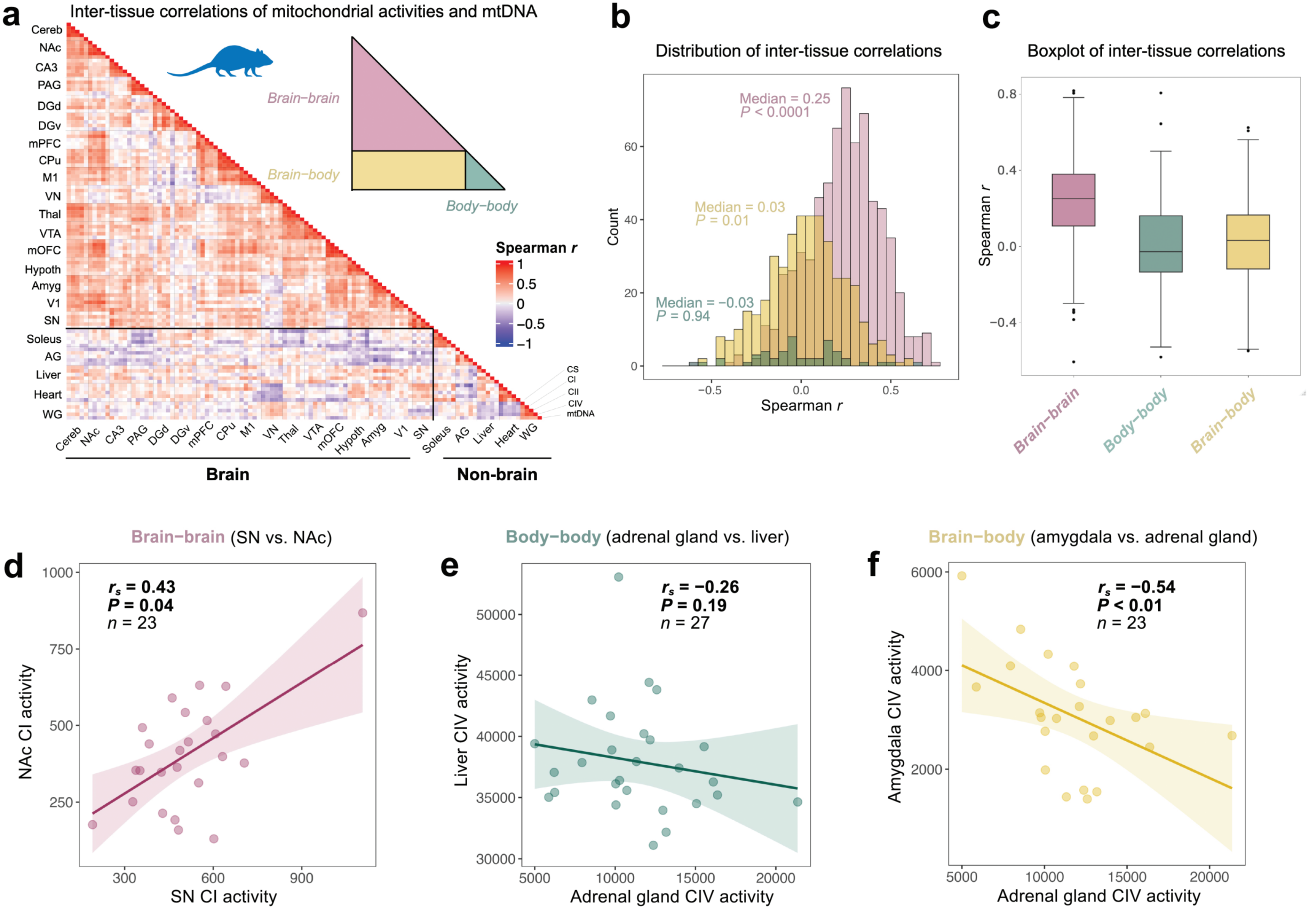
Mitochondrial enzymatic activity and mtDNA density measures display low between-tissue correlations across 22 tissues from 27 mice. (a) Enzymatic activity and mtDNA density-based multi-tissue mitochondrial distribution patterns displayed as correlation matrix of enzymatic activity measures (CI, CII, CIV, and CS) and mtDNA density across 17 brain tissues and five peripheral tissues from 27 male mice in Cohort 2 (*n* = 1,155 tissue pairs). (b) Frequency distribution of Spearman *r* correlation coefficients of pairwise tissue comparisons showing brain–brain (red), body–body (green), and brain–body (yellow) tissue comparisons. (c) Boxplot of brain–brain, body–body, and brain–body inter-tissue correlations. (d−f) Bivariate plots of mitochondrial enzyme activity measures between tissues. Abbreviations: Cereb, cerebellum; NAc, nucleus accumbens; CA3, CA3 region; PAG, periaqueductal gray; DGd, dorsal dentate gyrus; DGv, ventral dentate gyrus; mPFC, medial prefrontal cortex; CPu, caudoputamen; M1, primary motor cortex; VN, vestibular nucleus; Thal, thalamus; VTA, ventral tegmental area; mOFC, medial orbitofrontal cortex; Hypo, hypothalamus; Amyg, amygdala; V1, primary visual cortex; SN, substantia nigra; Soleus, red oxidative skeletal muscle; AG, adrenal gland; WG, white glycolytic skeletal muscle.

Intrigued by the consistency of these findings across multiple mitochondrial enzymes from several tissues, we replicated this analysis in a second cohort of 27 male mice, in which we quantified OxPhos enzyme activities and mtDNA density in 17 brain regions and 5 non-brain tissues (*n* = 1,155 tissue-tissue pairs), the largest multi-tissue mitochondrial biochemistry dataset available to our knowledge. The resulting correlation matrix containing all measures across all tissues shown in Fig. 2a is divided into brain–brain, brain–body, and body–body correlations (see inset Fig. 2a). Small (*r* = 0.1−0.3), moderate (*r* = 0.3−0.5), and some large (*r* > 0.5) correlations were observed between brain regions, reflecting a certain degree of coherence between different parts of the same organ (median *r* = 0.25; Fig. 2b–d). Of the brain–brain correlations, 86% were positive (19.7% significant, uncorrected *P *< 0.05) and 14% were negative (0.15% significant, uncorrected *P* < 0.05). In contrast, there was no coherence between brain–body tissues (median *r* = 0.03) and between various non-brain tissues (*r* = −0.03) (Fig. 2b–e). In fact, some correlations were even negative: animals with high amygdala complex IV activity tended to have lower adrenal gland complex IV activity (*r* = −0.54, uncorrected *P* < 0.01; Fig. 2f). These data indicate a potential functional tradeoff between some tissues, where organisms who have high mitochondrial expression in one tissue have low expression in another (discussed further below).

Nevertheless, contrary to the default hypothesis that correlations would be positive and indicate inter-tissue coherence, 93.4% of brain–body correlations were not statistically different from 0 (45% negative, 55% positive; only 3.3% were significantly positive, and 3.3% were significantly negative, uncorrected *P* < 0.05). The same was observed between non-brain tissues, where 84% of the tissue-tissue correlations were not statistically different from 0 (54% negative, 46% positive; 8% significantly negative, and 8% significantly positive), roughly indicating chance-level results.

Thus, animals with high OxPhos capacity and mtDNA content in one tissue do not tend to have high OxPhos capacity in other tissues, demonstrating an overall lack of coherence in mitochondrial biology between tissues of the same organism. Both mouse datasets are available in Supplementary File S1.

### Transcriptome-based mitochondrial profiling

**Figure 3:**
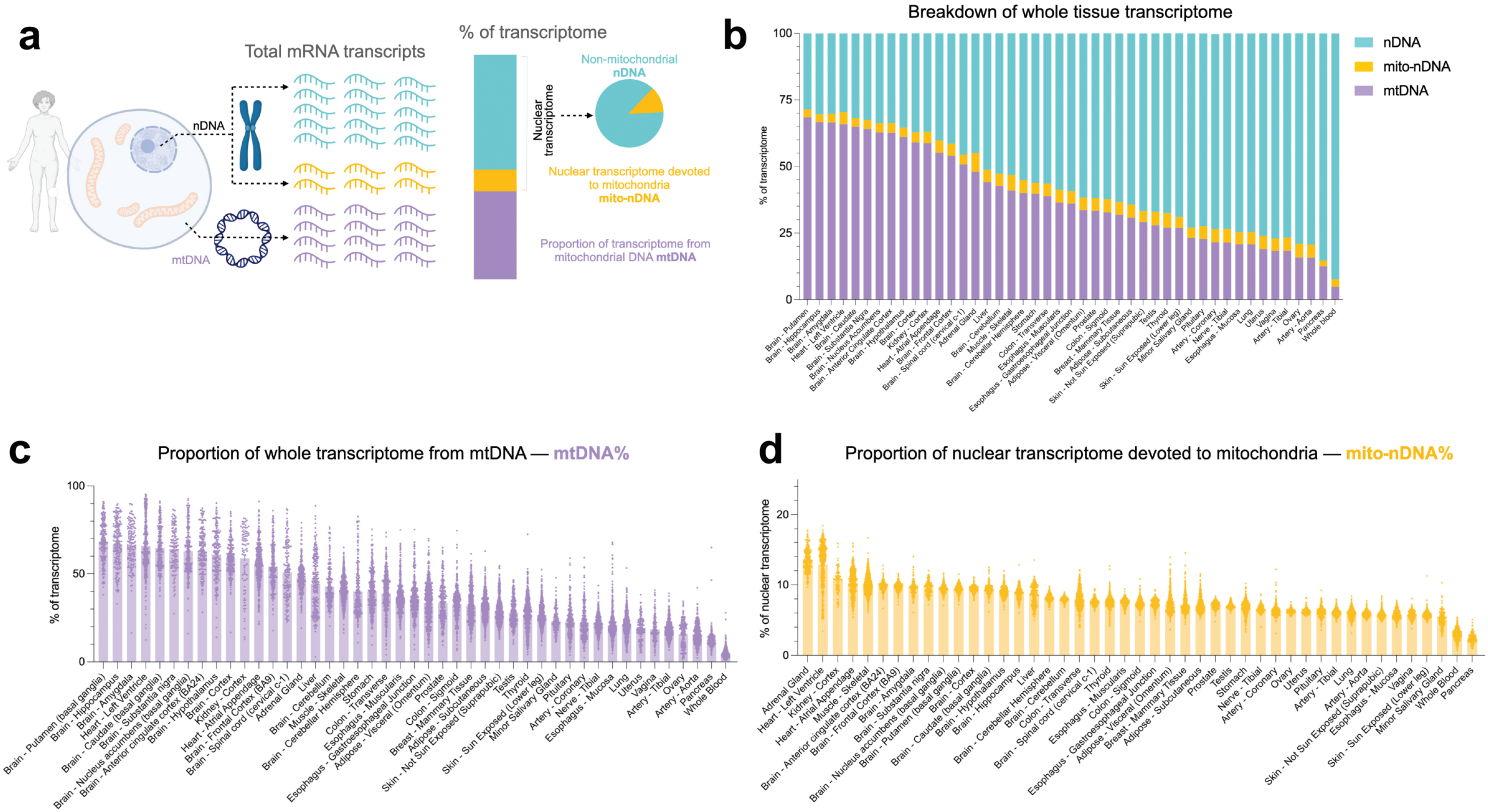
Transcriptome-based mitochondrial profiling across human tissues. (a) The percentage of total transcripts from the mtDNA, nuclear mitochondrial genes, and non-mitochondrial nuclear genes quantified in each sample (*n* = 16,205). (b) The average percentage of transcripts that are from mtDNA and nuclear mitochondrial genes (1120 genes Mitocarta 3.0) in each tissue. (C) Ranked mean percentage of total transcripts that are mtDNA transcripts in each tissue (each datapoint represents a person). (D) Ranked mean percentage of nuclear transcripts that are transcripts of mitochondrial nuclear genes (each datapoint represents a person).

Next, we examined the trait-level inter-tissue coherence hypothesis in humans. We leveraged the genotype-tissue expression (GTEx) [17] RNA sequencing (RNAseq) data from 948 women and men across 45 tissues (16,205 samples), which similarly allowed us to perform multiple brain–brain, brain–body, and body–body tissue comparisons. Using gene expression data for each tissue, we quantified the percentage of all mRNA transcripts that are derived from the mitochondrial genome (mtDNA%), reflecting most directly, albeit imperfectly, the mass or content of mitochondria within each person/tissue. Separately, based on the inventory of mitochondrial genes Mitocarta 3.0 [18], we quantified for each GTEx participant and tissue the proportion of the nuclear transcriptome (all nuclear transcripts arising from ~20,000 genes) that is “invested” to produce the ~1,100 mitochondrial proteins (the proportion of nuclear transcripts devoted to mitochondria [mito-nDNA%]). This yielded novel quantitative indices of mitochondrial regulation for each participant–tissue combination (Fig. 3a), available in Supplementary File S2.

As expected, the tissues with the highest fraction of mtDNA-derived transcripts (mtDNA%) were brain regions (putamen, hippocampus, and amygdala), followed by the heart (left ventricle), with mtDNA transcripts composing on average ~60%−70% of all mRNA transcripts (Fig. 3b). In a few individual participant’s brains and hearts, > 90% of mRNAs were of mtDNA origin. The lowest ranking tissues in mtDNA% were the pancreas and whole blood, where only ~4.8%–12.5% on average of the cellular transcriptome is from the mitochondrial genome (Fig. 3c). This result was expected as blood leukocytes, particularly the abundant neutrophils, have few mitochondria and few mtDNA copies per cell [19], and participants had on average 4.8% mtDNA% (ranged 0.4%–27.8%). The difference between the average of the highest and the lowest ranking tissues was 14.1-fold, reflecting the well-known natural variation in mtDNA copies and mitochondrial abundance between human tissues [13, 20].

The tissues with the highest mito-nDNA% were the adrenal gland and heart (left ventricle) (Fig. 3d), consistent with their high mitochondrial volume density [21, 22]. As in mtDNA-derived transcripts, the lowest ranking tissues for nuclear transcripts were whole blood and pancreas (Fig. 3d). As expected from well-established and stable tissue differences in mitochondrial content, tissues with higher mtDNA% also generally expressed higher mito-nDNA% (*r* = 0.84, *P* < 0.0001; Supplementary Fig. S2), lending validity to these measures. We analyzed the association of these parameters at the individual level within each tissue below.

### Human mitochondrial gene expression patterns display weak brain–body coherence

**Figure 4:**
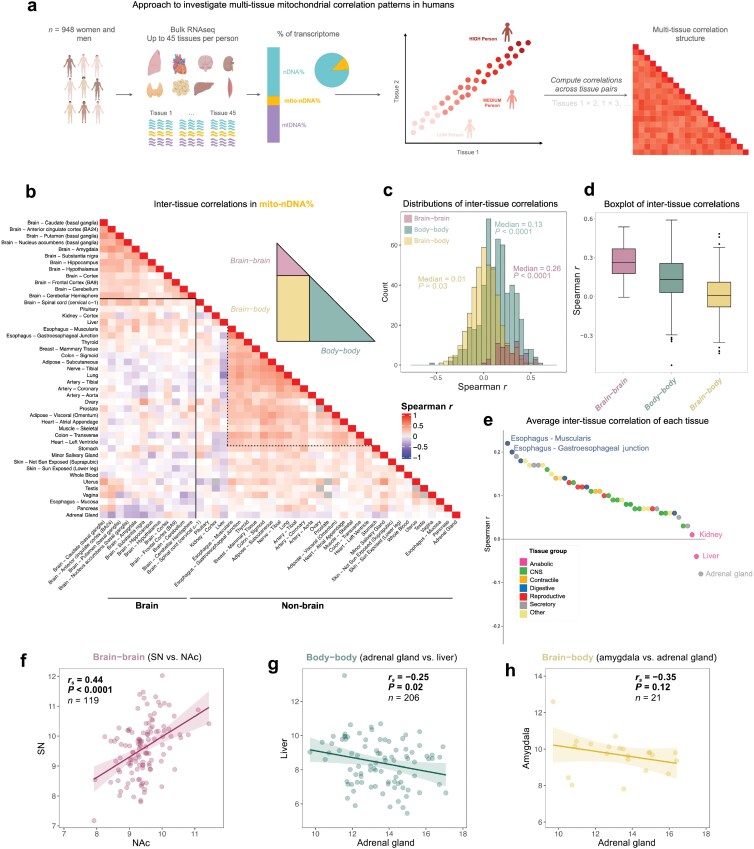
Mitochondrial gene expression patterns in human tissues display low between-tissue correlations. (a) Experimental set-up of multi-tissue correlation analysis of GTEx RNAseq data. (b) Heatmap of correlation matrix showing the pairwise Spearman *r* correlations of mito-nDNA% between 45 organs/tissues. The dotted lines highlight body tissues showing medium positive correlations. Grey cells in the heatmap indicate missing values. (c) Frequency distribution of Spearman *r* correlation coefficients (983 tissue comparisons) between brain–brain, body–body, and brain–body tissues. (d) Boxplot displaying median of Spearman *r* correlation coefficients of brain–brain, body–body, and brain–body tissue pairs. (e) Average inter-tissue correlation of each tissue. (f−h) Bivariate plots of mito-nDNA% between tissues.

Having established proximate markers of mitochondrial abundance and expression from RNAseq data, we then tested the inter-tissue coherence hypothesis in humans by quantifying mtDNA% and mito-nDNA% for all available GTEx subjects across 45 organs and tissues. We proceeded to compute the correlations across all tissue pairs (*n* = 983 tissue-tissue pairs) and analyzed the multi-tissue correlation structure (Fig. 4a). A heatmap representing the multi-tissue correlation structure of mito-nDNA% is shown in Fig. 4b, divided into brain–brain, body–body, and brain–body comparisons. The sample-size of each tissue comparison in the heatmap can be found in Supplementary Fig. S3.

The highest between-tissue correlations were observed between the 12 brain areas with a median of *r* = 0.26 (Fig. 4b). This result is similar to the median correlation (*r* = 0.25) observed between the 17 brain areas in mice (Fig. 2b). Correlations of body tissues with other tissues had a median of *r* = 0.13. As in mouse tissues, several human non-brain tissues showed medium positive correlation, indicating some coherence between specific tissues (highlighted with dotted lines; Fig. 4b), while others showed weak positive or even negative correlations. Again, the brain–body tissue pairs displayed minimal correlations (median *r* = 0.01; Fig. 4c and d). The adrenal gland exhibited the lowest degree of coherence with other tissues (mean *r* = −0.08), while the esophagus was most coherent with all other tissues (mean *r* = 0.22; Fig. 4e). As in mice, the brain substantia nigra and nucleus accumbens showed a medium positive correlation (*r* = 0.44, *P* < 0.0001; Fig. 4f), consistent with conserved cross-species neuroanatomical and functional connectivity. Also in agreement with our mouse cohort, the amygdala and the liver both showed negative correlations with the adrenal gland (Fig. 4g and h). Individuals with greater mito-nDNA% in their amygdala and liver tended to have lower adrenal gland mito-nDNA%, indicating minimal co-regulation and potentially the existence of tradeoffs between these tissues.

**Figure 5:**
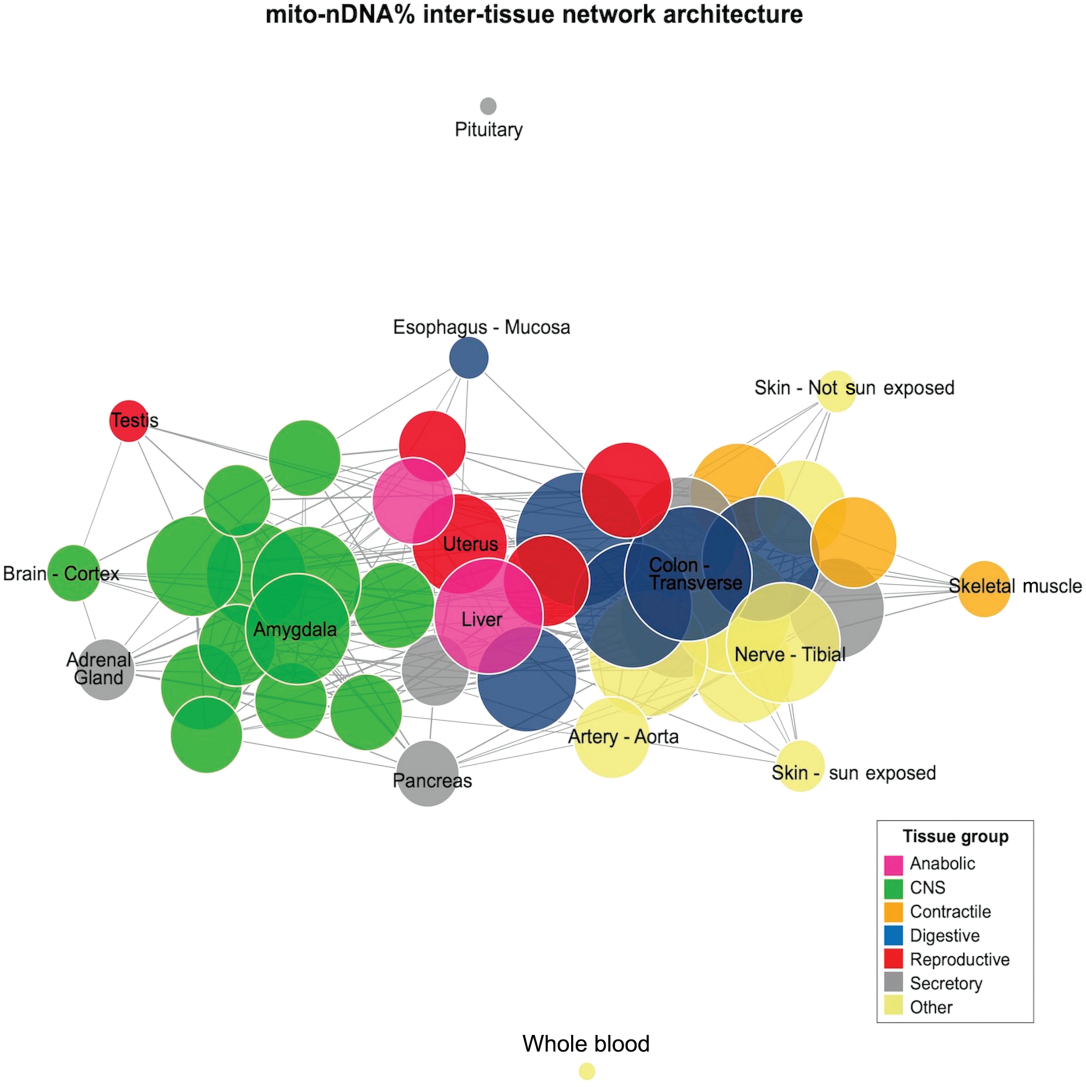
Multi-tissue network architecture of mitochondrial gene expression. Network representation of multi-tissue correlation matrix of mito-nDNA%. Each node represents a tissue, the size of each node is proportional to its degree, and the edge thickness is proportional to the strength of the correlation.

Repeating this analysis with mtDNA% of transcriptome, or with specific functional mitochondrial pathways, yielded similar results (Supplementary Fig. S4). While different mitochondrial pathways (specialized subsets of genes) exhibited relatively distinct correlation structures, all pathways exhibited medium correlations between brain regions and among some body tissues. However, regardless of the mitochondrial gene subsets selected, there was a uniform lack of coherence among brain–body tissue pairs, confirming the above results for mito-nDNA%. The multi-tissue network architecture of mitochondrial gene expression highlighting tissue pairs with the strongest and the weakest coherence is visualized in Fig. 5. The equivalent network for mtDNA% is presented in Supplementary Fig. S5. Tissues of the same type (e.g. brain regions, digestive tube segments) exhibit the greatest coherence, as expected, highlighting the tissue specialization of the mitochondria [23].

### The mtDNAcn-based inter-tissue correlation patterns are consistent with mitochondrial transcript-based patterns in humans

To validate our transcriptome-based findings indicating low coherence between brain and body tissues using a different method, we leveraged the recently reported qPCR-based catalog of mtDNAcn across 52 tissues from 952 GTEx subjects [20]. We asked whether individuals who have higher or lower mtDNAcn relative to other people in one tissue also have higher or lower mtDNAcn in other tissues. The resulting inter-tissue correlation structure of mtDNAcn across the same 45 tissues used in our transcript-based analyses is shown in Supplementary Fig. S6. The resulting correlation pattern was highly consistent with that observed using transcript-based indices of mitochondrial expression. Again, brain–body tissue pairs exhibited the lowest coherence (median *r* = −0.03). Body–body inter-tissue correlations had a median of *r* = 0.10. Again, the highest coherence was observed between brain tissues (median *r* = 0.24), with an effect size highly consistent with both the mouse (median *r* = 0.25) and human transcript-based indices of coherence (median *r *= 0.26). Thus, these results confirm the consistent yet modest coherence between regions of the same organ (brain) and the lack of coherence in mitochondrial biology across brain and body tissues.

### Low inter-tissue coherence at the proteomic level

We also tested the trait-level inter-tissue coherence hypothesis at the level of proteins using the same approach as above but with available proteomics data from a subset of the GTEx cohort: 201 samples from 32 tissues across 14 GTEx subjects (*n* = 19 tissue–tissue pairs after filtering the data) [24]. There was an insufficient number of brain samples in this dataset to perform brain–body inter-tissue correlation analyses. The resulting multi-tissue correlation structure and frequency distribution of correlation coefficients (Supplementary Fig. S7) agreed with the transcriptomics data, indicating no strong inter-tissue coherence. Several tissue pairs showed weak, and in some cases negative, correlations in mitochondrial protein abundance, meaning that an individual with high mitochondrial protein abundance in one tissue can have low abundance in others. Although the strength of the correlations is likely inflated due to the low sample size in this analysis, the median correlation coefficient (*r* = 0.26) from the proteomic data in non-brain tissues indicated potential modest inter-tissue coherence.

### Mitochondrial gene expression is driven in part by canonical energy sensing pathways

If mitochondrial content is not driven by a systemic trait-level factor, this raises the question: what determines the abundance of mitochondria in each tissue of an organism? We hypothesized that mitochondrial content may be driven by two potential pathways: (i) the transcriptional co-activator and master regulator of mitochondrial biogenesis peroxisome proliferator-activated receptor gamma coactivator 1-alpha (PGC-1α) [25], or (ii) the integrated stress response (ISR), which is a pathway that regulates energy balance and is induced by reductive stress and mitochondrial OxPhos defects in a tissue-specific manner [26, 27]. If correct, PGC-1α and ISR gene expression should exhibit positive intra-tissue correlations with mitochondrial gene expression.

**Figure 6:**
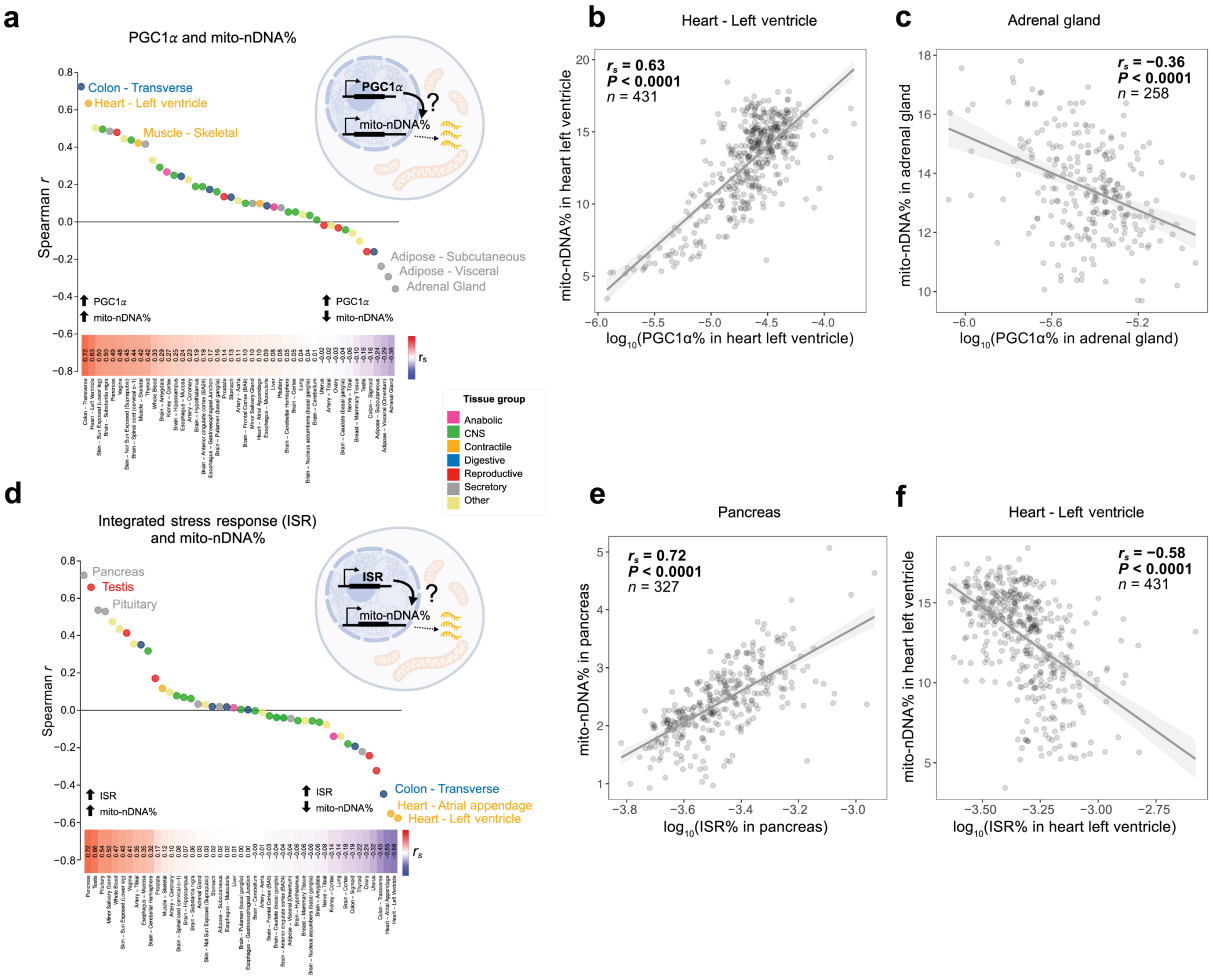
Mitochondrial gene expression is driven in part by canonical energy and stress-sensing metabolic pathways. (a) Ranked Spearman *r* correlation coefficients of PGC-1α% versus mito-nDNA% transcripts in each tissue. (b) Biplot showing the correlation of mito-nDNA% with PGC-1α expression in the heart (left ventricle). (c) Biplot showing the correlation of mito-nDNA% with PGC-1α expression in the adrenal gland. (d) Ranked Spearman *r* correlation coefficients of ISR% versus mito-nDNA% transcripts in each tissue. (e) Biplot showing the correlation of mito-nDNA% with ISR gene expression in the pancreas. (f) Biplot showing the correlation of mito-nDNA% with ISR expression in the heart (left ventricle).

The expression of PGC-1α was quantified by calculating the percentage of nuclear transcripts mapping to PGC-1α, and then correlating this to mito-nDNA% (Fig. 6a) or mtDNA% (Supplementary Fig. S8a), separately for each of the 45 tissues. As expected in the skeletal muscle and heart where PGC-1α was initially discovered to induce mitochondrial biogenesis [28, 29], individuals with high PGC-1α expression levels had significantly higher values of mito-nDNA%. This pattern was observed in 24 tissues (*P*_*s*_ < 0.05). The highest correlations were observed for the transverse colon and left ventricle of the heart (Fig. 6a and b). However, seven tissues exhibited significant negative correlations (*P*_*s*_ < 0.05), including the adrenal gland, visceral adipose, and subcutaneous adipose tissues where greater PGC-1α expression was related to lower mitochondrial gene expression (*r*_*s*_ = −0.24 to −0.36, *P*_*s*_ < 0.05) (Fig. 6a and c).

The ISR was quantified by calculating the percentage of nuclear transcripts mapping to four key mammalian ISR genes: activating transcription factor 4 (*ATF4*), *ATF5*, DNA damage inducible transcript 3 (*DDIT3*), and growth differentiation factor 15 (*GDF15*) [30–33]. Like PGC-1α, ISR gene expression was significantly associated with higher mito-nDNA%, but only in 12 tissues (*P*_*s*_ < 0.05; Fig. 6d). Interestingly, in 13 tissues, individuals with higher ISR activation exhibited lower mito-nDNA% (*P*_*s*_ < 0.05), demonstrating strong tissue specificity and a relatively equal number of tissues with positive and negative associations between ISR and mitochondrial gene expression (Fig. 6d–f). The strength and direction of the correlations between mitochondrial gene expression and either PGC-1α or the ISR genes were not significantly correlated (*r* = 0.02−0.18, *P* = 0.25−0.90), indicating that these pathways are not consistently co-regulated across human tissues.

Thus, if a nutritional, behavioral, or other stressor activated PGC-1α or ISR systemically, and if these pathways influenced mitochondrial gene expression, we would expect that some tissues would exhibit an increase, some tissue would not change, while others would exhibit a decrease in mitochondrial gene expression and/or biogenesis in response to the same stimulus. At the level of correlations, these organ-specific regulatory pathways would produce the lack of coherence and some apparent tradeoffs (high mitochondrial gene expression in some organs at the expense of low expression in others), as observed in our mouse and human cohorts.

For completeness, we also investigated other well-established regulators of mitochondrial biogenesis and maintenance. Using the same approach as above, we quantified the expression of nuclear respiratory factor 1 (*NRF1*), *NRF2*, mitochondrial transcription factor A (*TFAM*), and mitochondrial polymerase gamma (*POLG*) individually, and computed the correlation of each gene with mito-nDNA% and mtDNA% separately for each of the 45 tissues (Supplementary Fig. S9). Similar to PGC-1α and ISR genes, the results revealed highly tissue-specific relationships between mitochondrial gene expression and the genetic regulatory factors, with some tissues displaying strong/medium positive correlations and some negative. Each of the regulatory genes showed a distinct correlation pattern with mitochondrial gene expression across the 45 tissues, further highlighting the notion that mitochondrial gene expression and biology in different tissues may be driven by distinct molecular regulators.

### The discrepancy between mtDNA- and nuclear DNA (nDNA)-encoded transcripts is explained by tissue proliferation

The correlations of PGC-1α or ISR genes with mito-nDNA% were remarkably different from those with the marker of mitochondrial content, mtDNA% (Supplementary Fig. S8). For example, in the heart (left ventricle), whereas PGC-1α expression was positively correlated with the fraction of the nuclear genome devoted to mitochondria as expected (mito-nDNA%, *r* = 0.63, *P* < 0.0001; Fig. 6a), it was negatively correlated with the abundance of mtDNA-derived transcripts (mtDNA%, *r* = −0.36, *P* < 0.0001; Supplementary Fig. 8a). Brain tissues also exhibited this seemingly counterintuitive pattern where nDNA- and mtDNA-derived mitochondrial transcripts appear uncoupled or negatively correlated. Negative correlations between mtDNA- and nDNA-derived transcripts have been reported previously in human brain tissues from the GTEx cohort [34, 35]. The same pattern of opposite associations across certain tissues was observed for the ISR pathway (see Supplementary Fig. S8 for the heart as an example), suggesting that a cell-level or tissue-level factor is responsible for these tissue differences in the coupling of mtDNA- and nDNA-derived transcripts.

**Figure 7:**
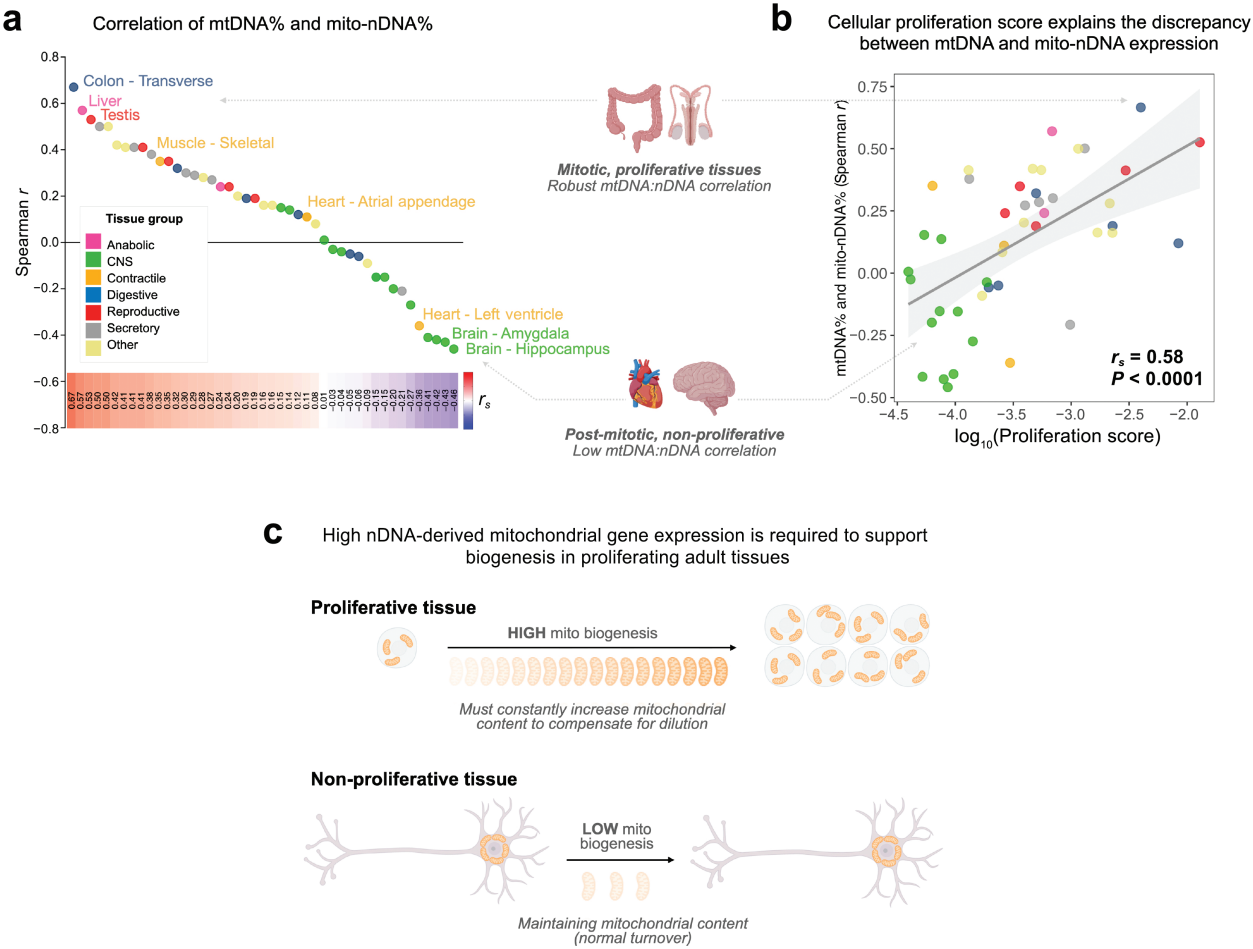
Discrepancy between mtDNA and mito-nDNA transcripts is explained by proliferation gene expression. (a) Ranked Spearman *r* correlation coefficients of mtDNA% and mito-nDNA% transcripts in each tissue. (b) Biplot of proliferation score (average of *KI67*, *TOP2A*, and *RRM2*) and Spearman correlation coefficient of mtDNA% versus mito-nDNA% in each tissue. (c) Illustration of mitochondrial biogenesis in proliferative and non-proliferative tissues. In non-proliferative tissues that are also rich in mitochondria, such as the heart and brain, transcripts for nDNA-encoded mitochondrial proteins are relatively low as biogenesis is not prioritized, nevertheless mtDNA-encoded transcripts remain high due to the abundance of mitochondria. However, in actively proliferating tissues, such as the colon and testis, mitochondrial biogenesis is upregulated, resulting in high mito-nDNA and mtDNA transcripts to replenish the regular dilution of mitochondria in dividing cells.

Based on (i) the dilution of mitochondria occurring during cell division in proliferating tissues (digestive tract or reproductive organs) [36, 37], and (ii) the striking stability of mitochondrial proteins in non-replicative tissues (e.g. brain, heart, and muscle) [38, 39], we reasoned that this discrepancy between mtDNA- and nDNA-encoded transcripts and their associations with drivers of biogenesis could be attributable to tissue-specific differences in proliferative activity. Indeed, tissue proliferation (indexed by the average expression of three key proliferation genes: *KI67*, topoisomerase IIα (*TOP2A*), and ribonucleotide reductase regulatory subunit M2 (*RRM2*)) [40, 41] showed that the correlation of mtDNA% versus mito-nDNA% was positively associated with proliferation (Fig. 7a and b). In highly proliferative tissues (where newly made mitochondria are rapidly diluted by cell division; Fig. 7c, bottom), tissues investing a large portion of their nuclear transcriptome into mitochondrial biogenesis also have high mtDNA-derived transcripts (positive correlation), likely as a means to replenish the ~50% dilution that regularly takes place in symmetrically dividing cells. On the other hand, non-proliferative tissues with high mitochondrial mass tend to have proportionally lower nDNA-derived transcripts than the abundant mtDNA-derived transcripts contained in their numerous cytoplasmic mitochondria, consistent with the ability to maintain mitochondrial mass without sustained nuclear mitochondrial biogenesis in post-mitotic tissues (Fig. 7c, top). Tissue replicative activity therefore accounts for the relative abundance of mtDNA- and nDNA-derived mitochondrial transcripts across human tissues.

### Sub-groups of individuals display distinct multi-tissue mitochondrial distribution patterns

**Figure 8:**
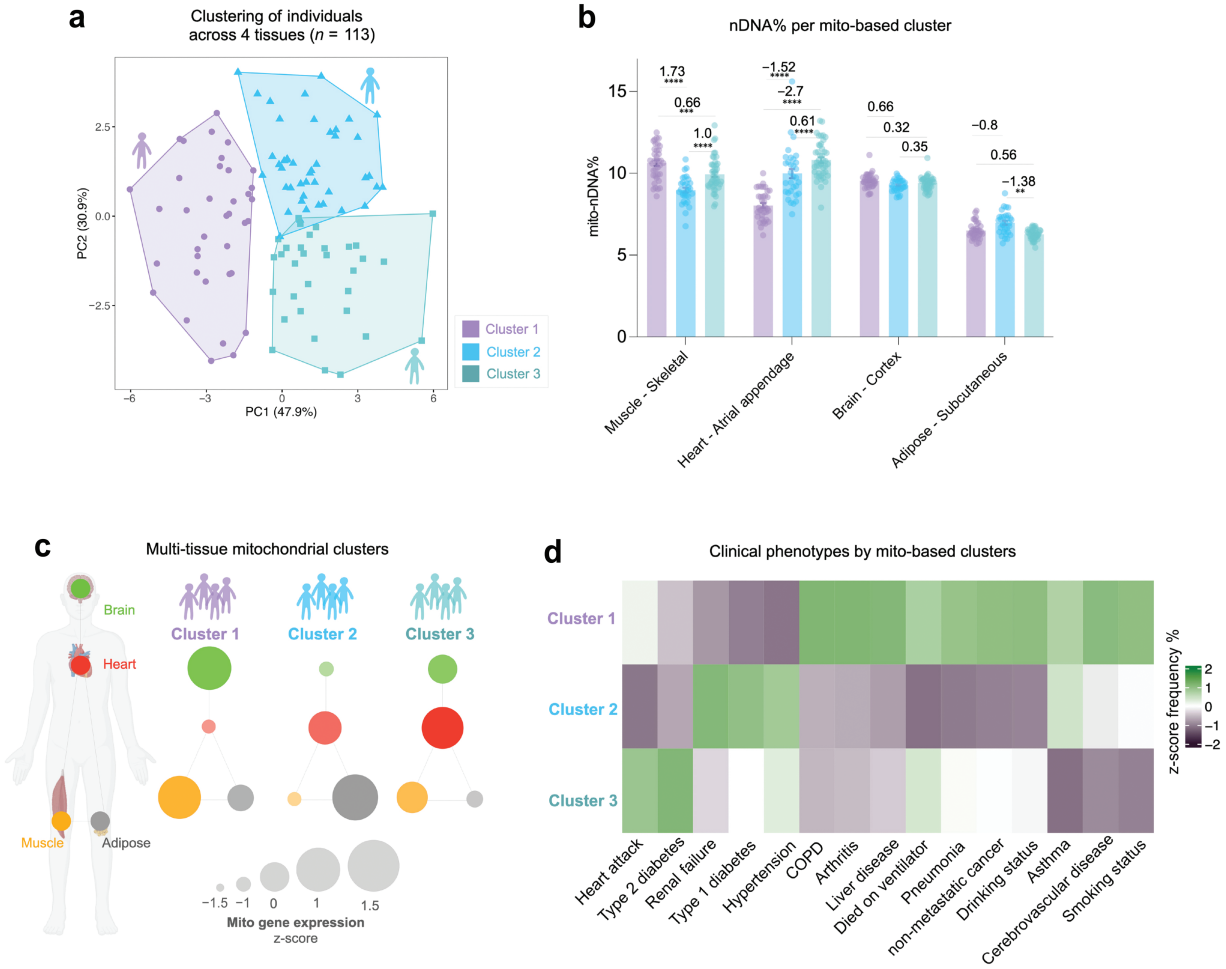
Sub-groups of individuals display different mitochondrial distribution patterns. (a) The k-means clustering on mito-nDNA% ratios between four tissues (muscle—skeletal, heart—atrial appendage, brain—cortex, adipose—subcutaneous tissue) from 113 subjects. Cluster 1, *n* = 37; Cluster 2, *n *= 32; Cluster 3, *n* = 44. (b) Bar plot of mean mito-nDNA% in each cluster across the four tissues. Cluster means of each tissue were tested for significant differences by two-way ANOVA. Effect sizes were computed by Hedge’s *g*. (c) Network visualization of *z*-score transformed mito-nDNA% of each cluster across the four tissues analyzed. (d) Heatmap showing the *z*-score percentage of subjects in each cluster recorded as positive for each clinical variable.

Our analysis of the multi-tissue correlation structure in GTEx showed that individuals can have a high mitochondrial investment in some tissues, but low in other tissues. Based on the notion that different organs age at different rates in different individuals [14–16], this led us to examine whether there are sub-groups or clusters of individuals who exhibit distinct multi-tissue mitochondrial distribution patterns. To address this question, we performed k-means clustering on mitochondrial gene expression data from 113 subjects with complete data across four tissues, interrogating the ratios in mito-nDNA% (Fig. 8a) and mtDNA% (Supplementary Fig. S10) between each tissue pairs: brain (cortex), heart (atrial appendage), muscle (skeletal), adipose (subcutaneous) (Fig. 8a).

Principal component analysis (PCA) of these data showed that the first two principal components accounted for 78.8% of the variance, from which three clusters of GTEx participants emerged. A two-way analysis of variance (ANOVA) on the mito-nDNA% values for each tissue confirmed that the identified subgroups of individuals exhibited significant differences in mitochondrial transcript abundance across three of the four tissues analyzed (Fig. 8b and c). The mitochondria-defined subgroups differed on clinical and phenotypic profiles, including causes of death and known medical diagnoses at the time of death (Fig. 8d). This association between multi-tissue mitochondrial profiles and clinical phenotypes provides preliminary evidence that distinct mitochondrial distribution strategies among the multi-organ network may be associated with different resilience or vulnerability profiles to disease.

## Discussion

Here, we tested the hypothesis that the regulation of mitochondrial biology among dozens of organs and tissues is driven by an individual trait-level factor. We addressed this question by examining the multi-tissue correlation structure among direct enzymatic measurements of mitochondrial OxPhos respiratory chain enzyme activities in two cohorts of mice, and from the transcriptome and mtDNAcn of 45 human tissues. Contrary to the default hypothesis that mitochondrial content is a trait-like, person-level attribute exhibiting high coherence across the organism, our results show a striking spectrum of mitochondrial distribution patterns. In particular, we found that individuals who have high markers of mitochondrial content in some tissues can have low markers of mitochondrial content in other tissues. These differences may be partially explained by the energy-sensing pathways, PGC-1α and ISR, as well as by proliferative activity of different tissues, expanding our understanding of the factors that may regulate the highly heterogenous and tissue-specific regulation of mitochondrial biology in humans and other animals. These results emphasize that genetic and systemic factors are insufficient to explain the mitochondrial expression patterns across the brain and body, and instead suggest that different individuals display relatively idiosyncratic patterns of mitochondrial regulation.

The results of our investigation into the inter-tissue correlation structure of mitochondria in mouse and human tissues are consistent with the idea that there is a partial coherence between the organs/tissues within an individual. This indicates that there is a certain degree of inter-tissue coregulation of mitochondria, particularly between tissues of the same type. We observed distinct distributions in coherence between brain–brain, body–body, and brain–body tissues. Different brain regions showed the highest coherence. However, there was minimal evidence of coherence between brain and body tissues. The specific tissues with the highest average coherence were esophagus-gastroesophageal junction and esophagus-muscularis. Adrenal gland, liver, and kidney displayed the lowest average inter-tissue coherence. Overall, given that the highest correlations of mito-nDNA% did not exceed *r* = 0.6, and many tissues showed no evidence of coherence, this indicates that every person may have an idiosyncratic mitochondrial distribution pattern, contributing to the uniqueness of each individual.

Our analysis of the relationship between mitochondrial gene expression and energetic stress sensing metabolic pathways (PGC-1α and ISR) revealed a strong tissue-dependent association. The results for PGC-1α were particularly striking. While consistent with previous evidence in skeletal muscle and heart, it appears that PGC-1α does not drive mitochondrial biogenesis in all tissues at the transcript level. In fact, an opposite association was observed in several tissues, calling for a more refined understanding of the forces that regulate mitochondrial biology and biogenesis, particularly in different organ systems. The novel association of mtDNA:nDNA ratio with proliferative activity across tissues provides an example of such a force that governs mitochondrial biogenesis in a tissue-specific manner.

The significant differences in mitochondrial gene expression patterns between subgroups of individuals suggest that some individuals “invest” more of their energetic resources in some tissues compared to others. This aligns with recent studies identifying distinct ageotypes (i.e. aging phenotypes or strategies) among individuals [14, 16, 42]. In our data, these groups also displayed different patterns of clinical phenotypes, opening the possibility that distinct mitochondrial distribution patterns among the multi-organ network may be associated with differences in resilience or vulnerability to specific diseases. This notion aligns with an emerging systemic understanding of mitochondrial biology with regulatory signals and even whole mitochondria transferred between organ systems and cell types [2, 43]. It remains an open question whether these clusters are permanent traits of an individual (determined, for example, by genetics or developmental conditions), or are adjusted temporally within an individual based on current conditions.

## Limitations of the study

An important limitation of the human portion of this study is the nature of postmortem tissue-derived data. In GTEx, postmortem interval time is associated with tissue-specific changes in transcript abundance [44]. This could have affected the transcriptome-based mitochondrial profiling and inter-tissue correlation analyses using GTEx data, but not the human mtDNAcn-based analyses nor the enzymatic and mtDNA-based measures in mouse tissue. Although the fraction of mtDNA transcripts is not a measure of sample quality in bulk RNAseq data as it is in single-cell analyses, we cannot rule out the possibility that the high percentage of mtDNA transcripts observed in some samples/tissues could reflect some poorly understood biological or technical confound. Only partially addressing this point, our sensitivity analyses showed that our transcriptome-based inter-tissue correlation patterns were stable across various RNA integrity number (RIN) cutoffs. Another limitation is that not all tissues were available for all individuals, limiting the number of tissue-tissue pairs available for some pairs of organs and tissues. Future studies with complete data in greater numbers of tissues and individuals, and with direct measures of mitochondrial functions (respiration, OxPhos, or ROS production), would be useful in extending and validating these results. We also note that the GTEx dataset includes a majority of White individuals, and therefore lacks representation of other ethnic groups, also possibly limiting generalizability. Finally, in mouse Cohort 2, animals were aged 52 weeks, representing animals in mid-life and therefore relatively well-matched to GTEx participants. However, this contrasts with the typical juvenile ages (8−12 weeks old) used in research, possibly limiting direct comparisons with previous studies.

## Methods

### Mouse tissue homogenization, enzymatic activity assays, and qPCR

Enzymatic activity assays and qPCR were performed on mouse tissues as described previously [45]. The first mouse cohort included 16 c57bl/6J female and male mice, from which five tissues were sampled, as described in [46]. The second mouse cohort (*n* = 27 mice, 22 tissues) is described in [45]. All mice in Cohort 2 were 52-week-old male c57bl/6J animals. The mtDNA density was quantified as described in [45]. Raw data was used to compute inter-tissue correlations for each mitochondrial feature including Complex I activity, Complex II activity, Complex IV activity, CS activity, and mtDNA density. A total of 50 (10 per mitochondrial feature) comparisons were computed for Cohort 1, and 1,155 (231 per mitochondrial feature) for Cohort 2.

### GTEx dataset

The GTEx v8 RNAseq dataset consists of 17,382 samples across 54 tissues from 948 donors. The GTEx cohort is 67.1% male and 32.9% female, among which 84.6% are White, 12.9% are African American, 1.3% are Asian, and 1.1% are others. The age range of donors is 20−70 years (mean ± SD = 52.8 ± 12.9 years). Further information on the GTEx v8 dataset can be found on the GTEx portal at https://gtexportal.org/home/tissueSummaryPage.

### Transcriptomics

RNAseq data was downloaded as transcripts per million (TPM) from the GTEx portal. Data is available for download at https://gtexportal.org/home/downloads/adult-gtex/bulk_tissue_expression. Subject clinical phenotype data is part of the protected access data and was obtained through the dbGAP accession #phs000424.v8.p2 under project #27813 (Defining conserved age-related gene expression trajectories). All samples with an RIN value of less than 5.5 were filtered out of the data. For inter-tissue correlation analysis, a minimum sample size of 10 shared subjects was set for each tissue pair and all tissue pairs with less than 10 subjects were not included in the analysis. Tissues that did not meet this shared sample size with more than 50% of other tissues were not included in the analysis. Cell lines were also removed from the dataset. After applying these filters to the data, 45 tissues and 16,205 samples remained and were included in the analysis. The mitochondrial genes included in analyses were the Mitocarta 3.0 genes (of which we identified 1133 in the dataset) and all other mtDNA genes. TPM values were normalized separately for mtDNA and nDNA genes. The mtDNA genes were expressed as a percentage of all transcripts in a sample. Nuclear genes were normalized by removing all mtDNA transcripts from the sample, and then expressed as a percentage of all remaining transcripts (proportion of nuclear transcriptome). We performed a sensitivity analysis by repeating our mito-nDNA% inter-tissue correlation analysis with a sample RIN cutoff of RIN ≥ 6 and RIN ≥ 7. This had a negligible effect on the median inter-tissue correlation and correlation structure, but it greatly reduced the number of tissue pairs that could be included in the analysis. Therefore, we chose a less stringent RIN cutoff of 5.5 to maximize inter-tissue sample sizes and the number of tissue pairs included in our analysis.

For the analyses of potential regulators of mitochondrial gene expression, we used the percentage of nuclear transcripts mapping to either PGC-1α (*PPARGC1a*), ISR genes (*ATF4*, *ATF5*, *DDIT3*, and *GDF15*), or additional molecular regulators of mitochondrial gene expression, biogenesis, and mtDNA replication separately (*NRF1*, *NRF2*, *TFAM*, and *POLG*).

### Proteomics

Proteomics data were obtained from the Supplementary Information in Jiang *et al*. 2020 [24]. The “protein normalized abundance” in Supplementary Table S2c was downloaded and used for analysis. The abundances of mitochondrial genes, of which we identified 1002, were summed and expressed as a percentage of total protein abundance to establish a proximate marker of mitochondrial abundance in each sample. Replicate measurements of samples from multiple proteomics runs were averaged for the pairwise inter-tissue correlation analysis. Only tissue pairs with a minimum of eight shared subjects were included in the analysis. Tissues that did not meet this minimum sample size with at least four other tissues were excluded. This resulted in seven tissues and 14 subjects included in the analysis.

### The mtDNAcn data analysis

The mtDNAcn data on the GTEx cohort were downloaded from the supporting information in Rath *et al*. 2024 [20]. The data were filtered for the same 45 tissues used in transcriptomics data analysis. Only tissue pairs with a minimum of 10 shared subjects were included in the analysis. Individual mtDNAcn values for each GTEx tissue sample were used for inter-tissue correlation analysis.

### Tissue proliferation index

Our criteria for selecting genes to include in the proliferation index were as follows: (i) the genes are known in the literature to be markers of proliferation [40, 41]; (ii) the genes must display high expression in tissues that are proliferative (e.g. blood and digestive tract) [36]; (iii) the genes must show internal consistency (i.e. positively correlated within tissues); and (iv) the genes must display high expression during periods of rapid cell division in a human primary tissue culture system [47]. The genes selected for the tissue proliferation index were *KI67*, *TOP2A*, and *RRM2*. The index was established by averaging the expression of these three genes in each tissue sample.

### Multi-tissue network graph

The multi-tissue network graph in Fig. 5 was generated with the R package iGraph. An adjacency matrix was generated from the inter-tissue correlation matrix shown in Fig. 4b, setting the edge threshold to *r* = 0.2. The layout of nodes was determined by the Fruchterman-Reingold layout force-directed algorithm.

### Statistics

Statistical tests were performed with R version 4.4.0 (2024-04-24) and GraphPad Prism version 10. The correlations between pairwise tissue comparisons were assessed using Spearman rank correlation. Clustering was performed on inter-tissue ratios using k-means method and was visualized using PCA. Two-way ANOVA was used to test mitochondrial gene expression of identified clusters for significant differences. Effect sizes were estimated using Hedge’s *g*. The significance level for *P*-values was set at *P* < 0.05.

## Supplementary Material

loaf012_suppl_Supplementary_Figures_S1-S10

loaf012_suppl_Supplementary_File_S1

loaf012_suppl_Supplementary_File_S2

## Data Availability

The GTEx v8 RNAseq dataset can be downloaded from the GTEx portal. All R code used in the analyses can be found at github.com/mitopsychobio.
