# Supplementary material for: Brain–body mitochondrial distribution patterns lack coherence and point to tissue-specific regulatory mechanisms
Source: Life Metab. 2025 Apr 12;4(3):loaf012. doi: 10.1093/lifemeta/loaf012 (PMC12141818; doi:10.1093/lifemeta/loaf012)
Supplement: loaf012_suppl_Supplementary_Figures_S1-S10 [file loaf012_suppl_supplementary_figures_s1-s10.pdf]

Supplementary Figure S1

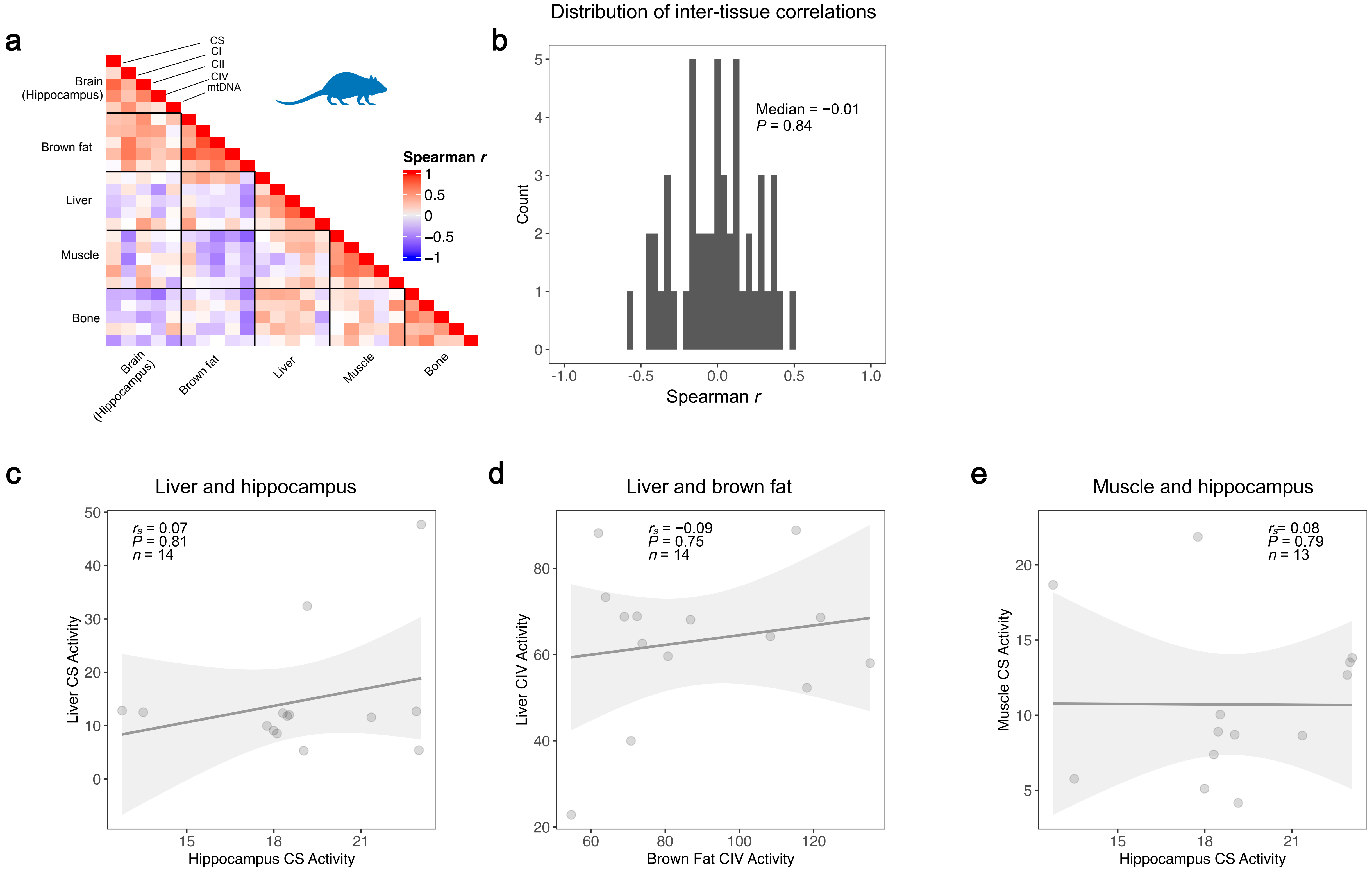

Supplementary Figure S2

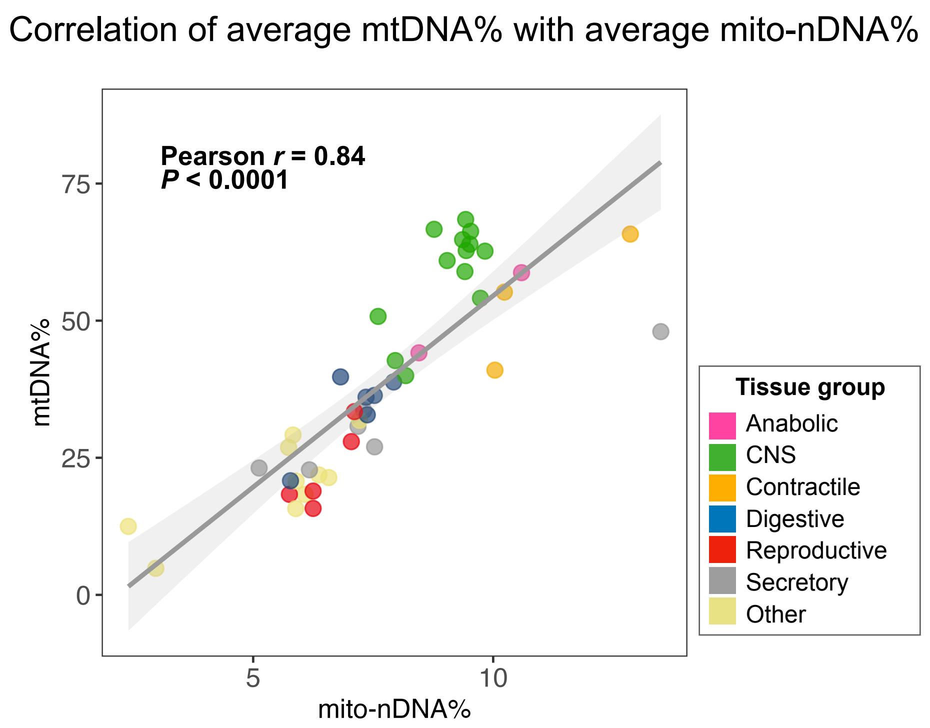

Supplementary Figure S3

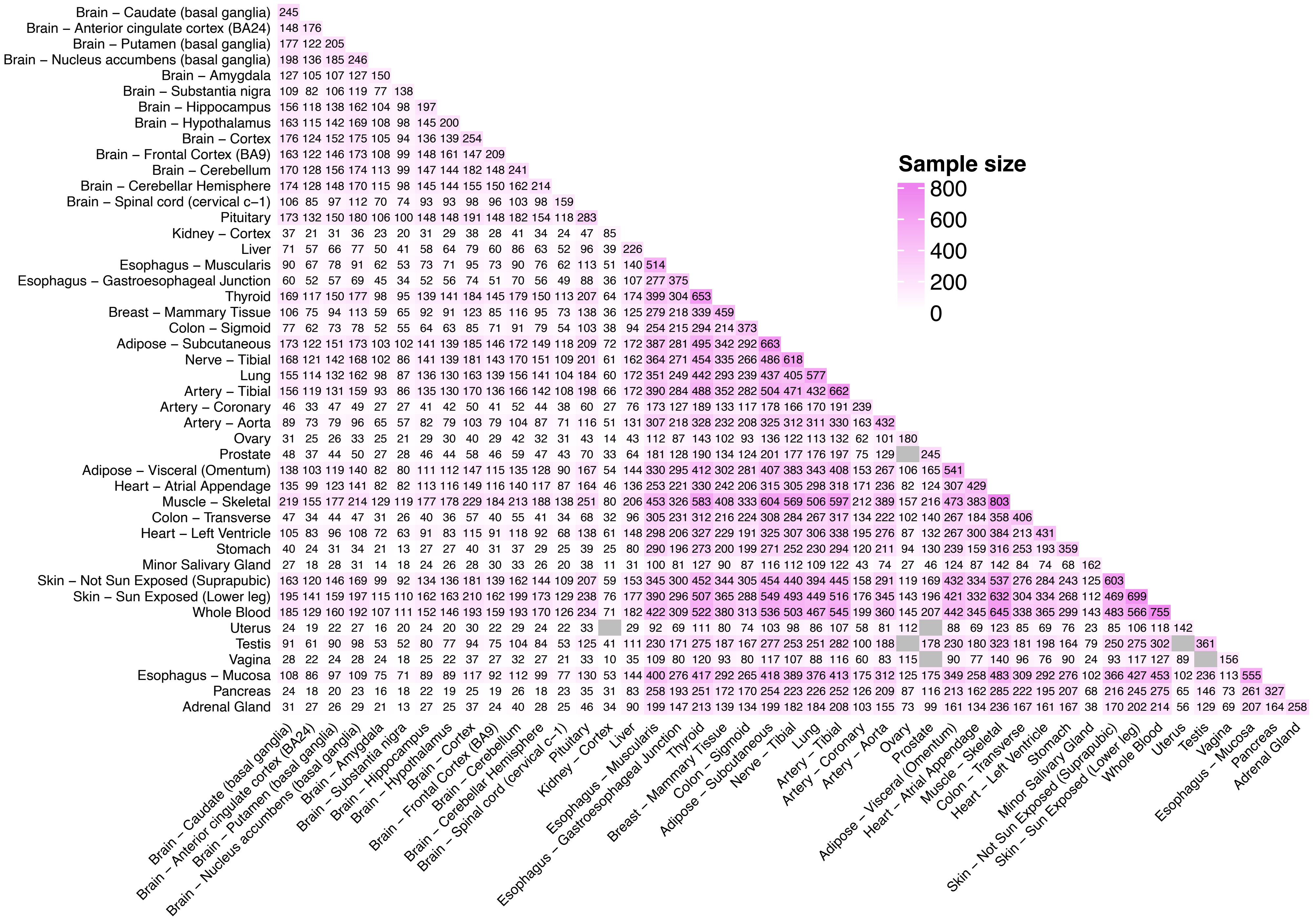

Supplementary Figure S4

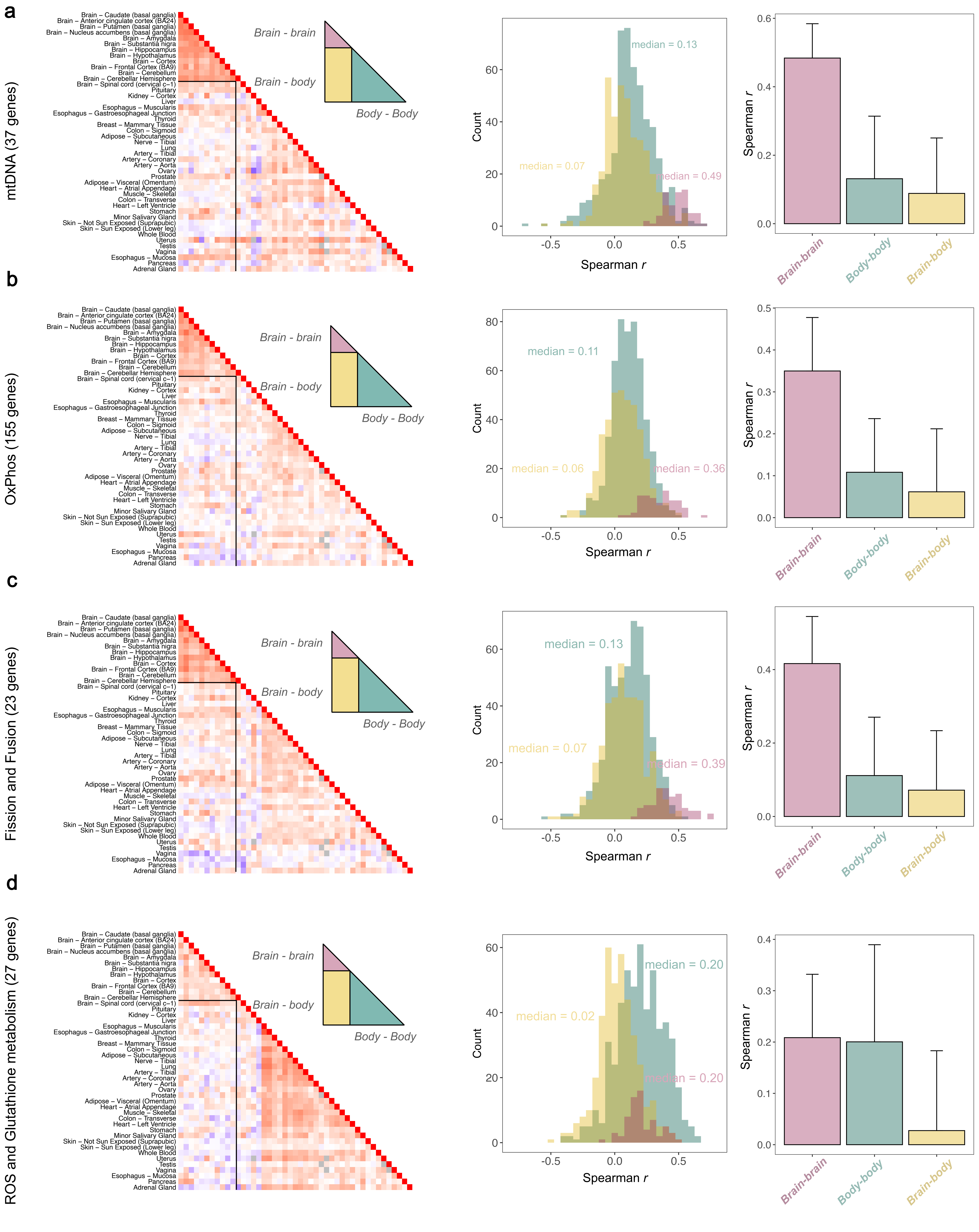

Supplementary Figure S5

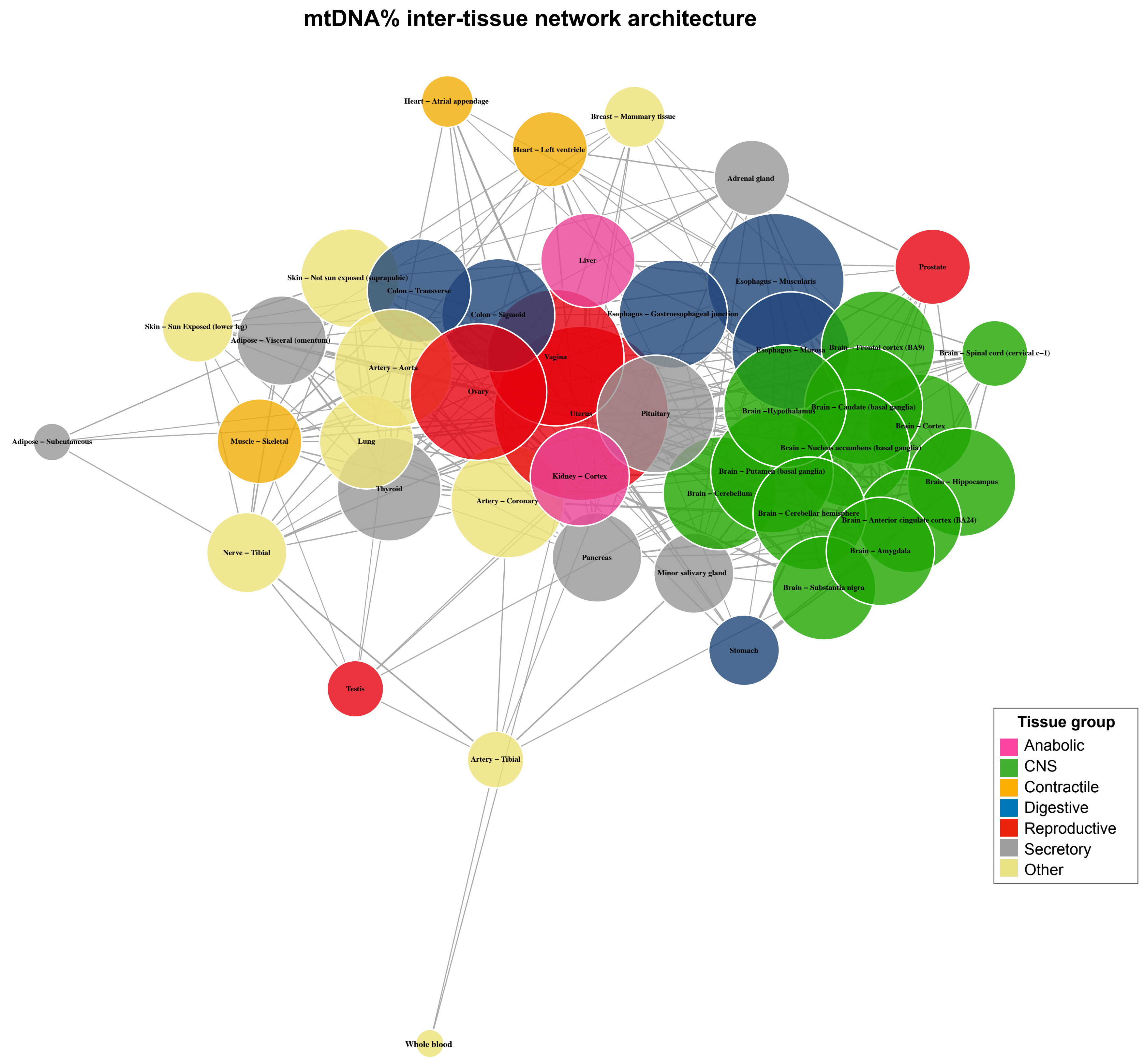

Supplementary Figure S6

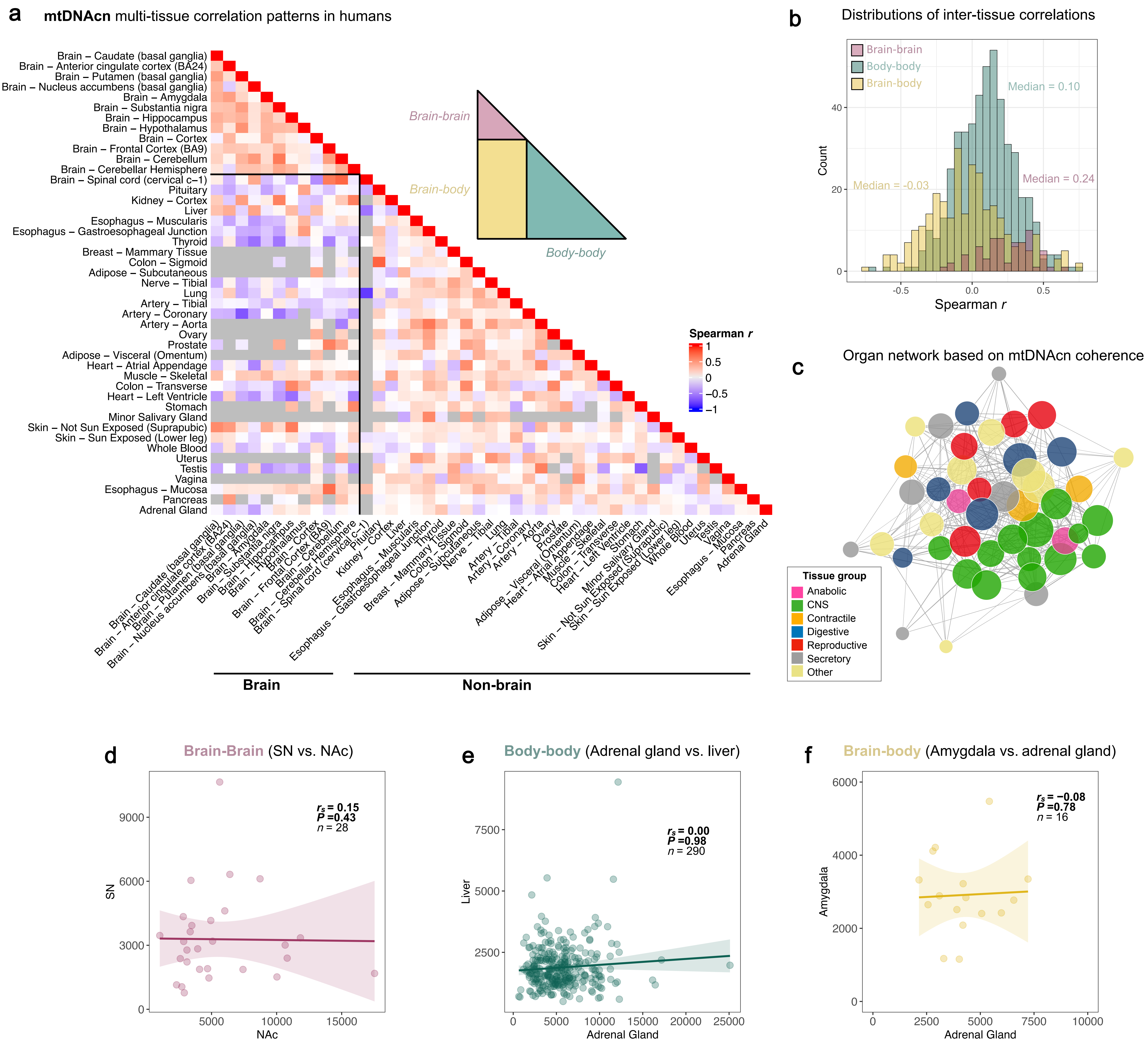

Supplementary Figure S7

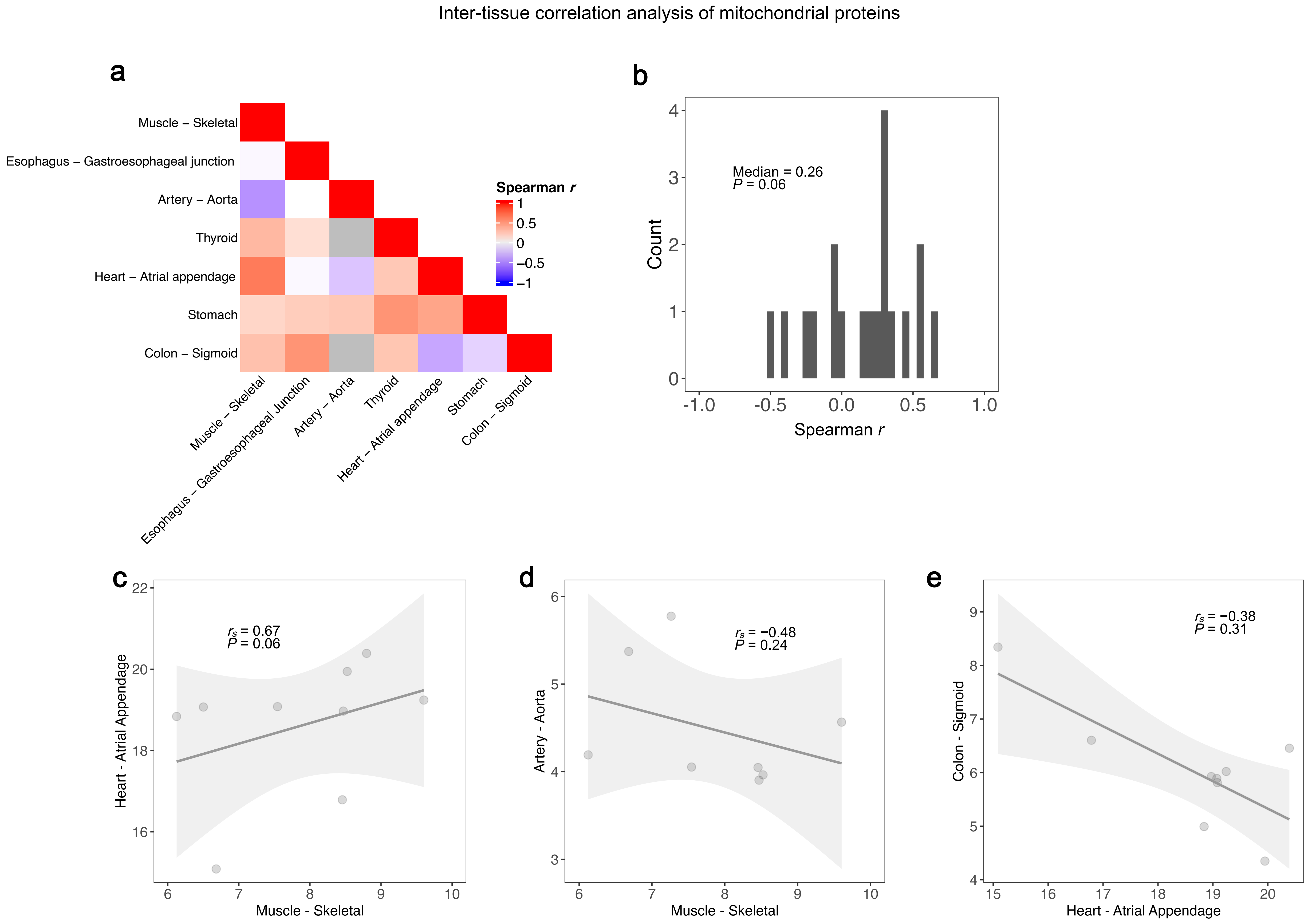

Supplementary Figure S8

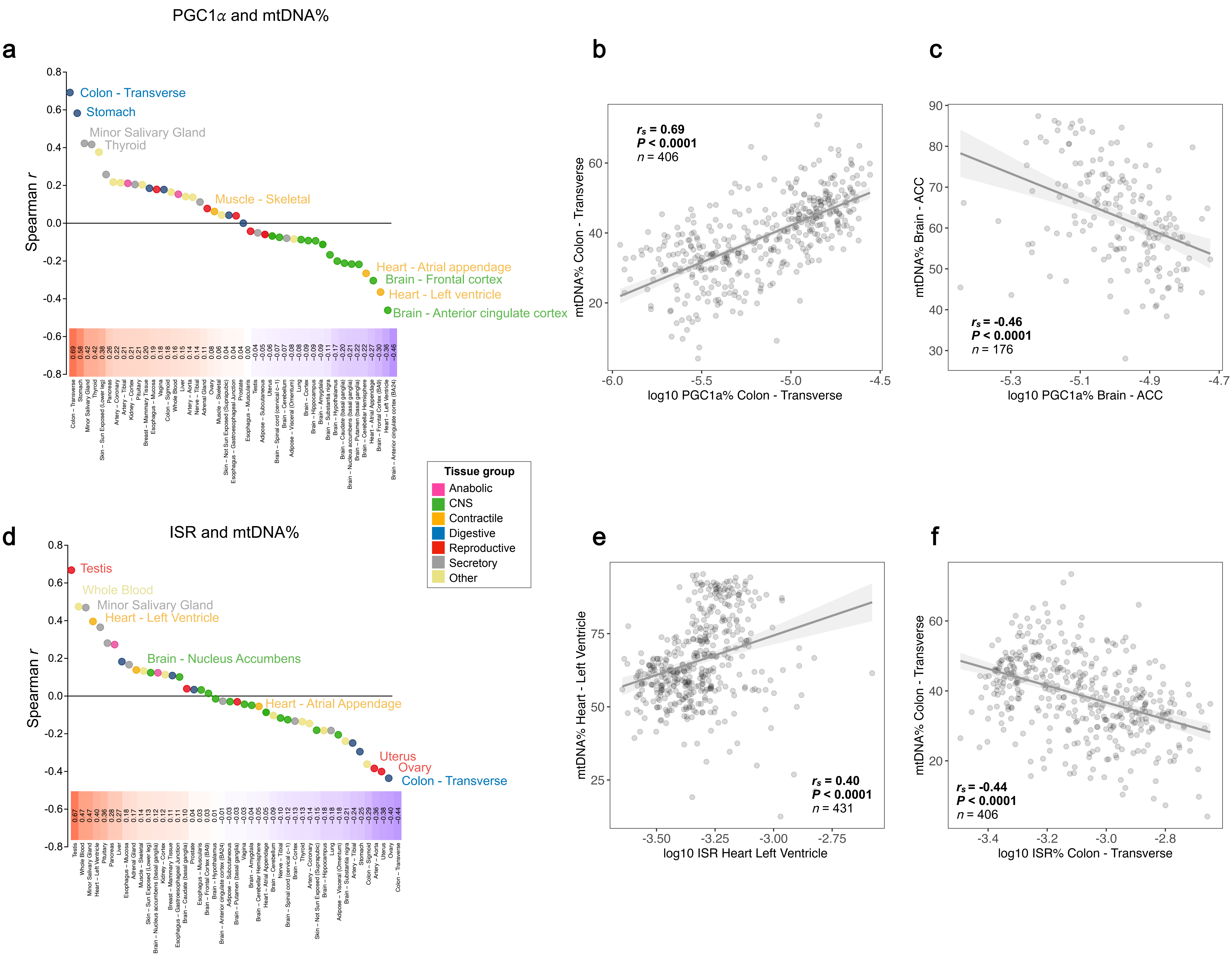

Supplementary Figure S9

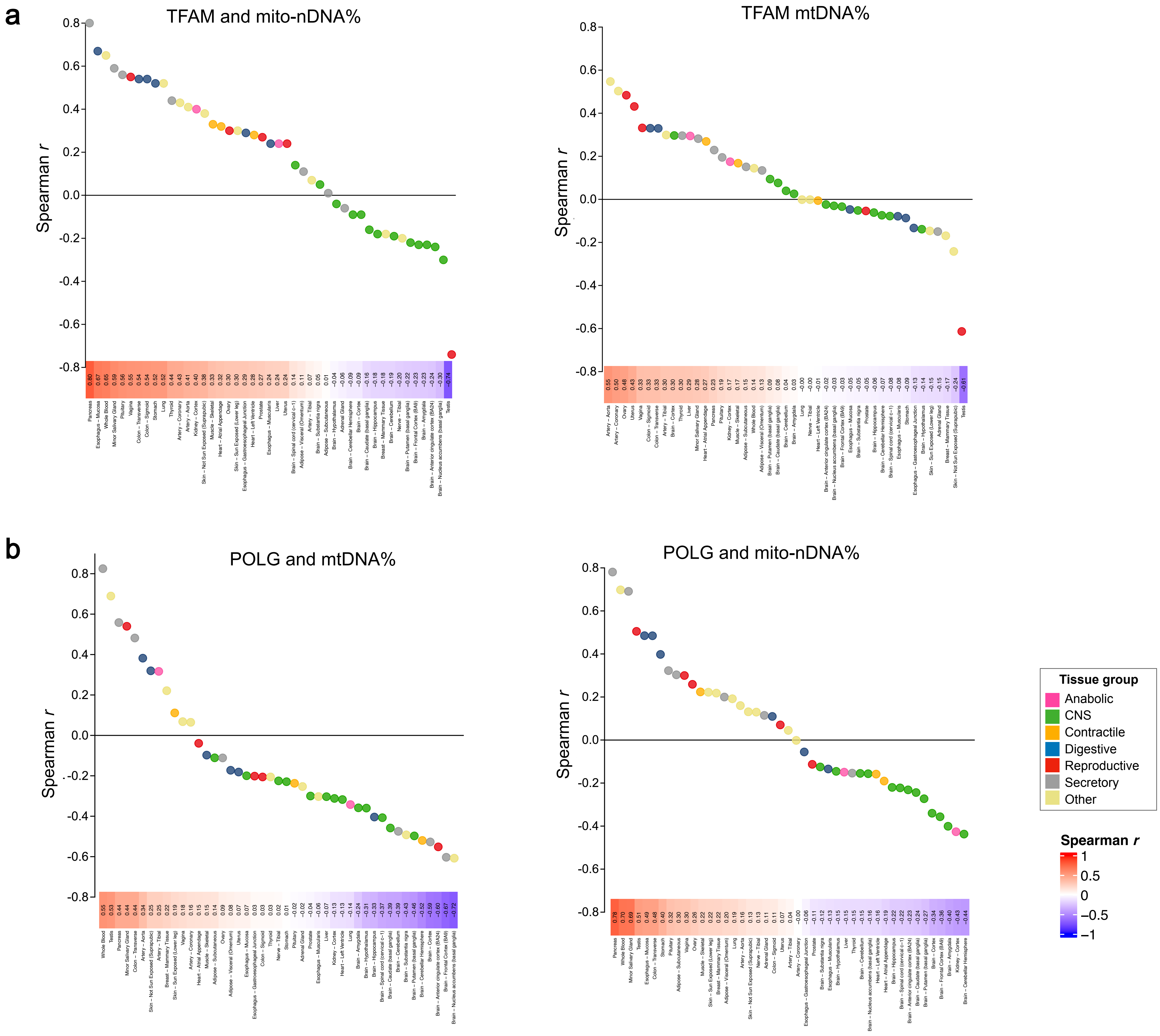

Supplementary Figure S9 continued

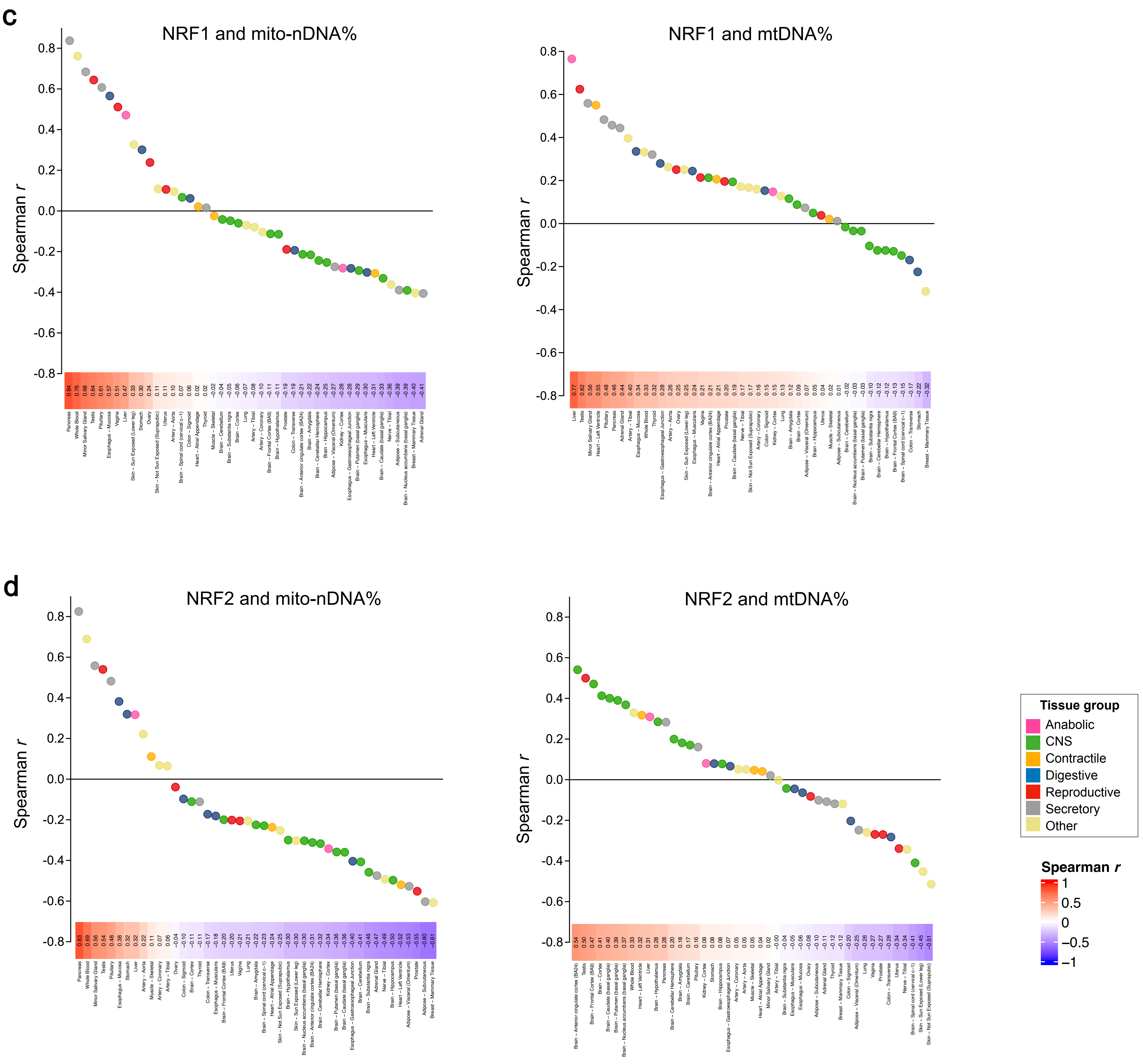

Supplementary Figure S10

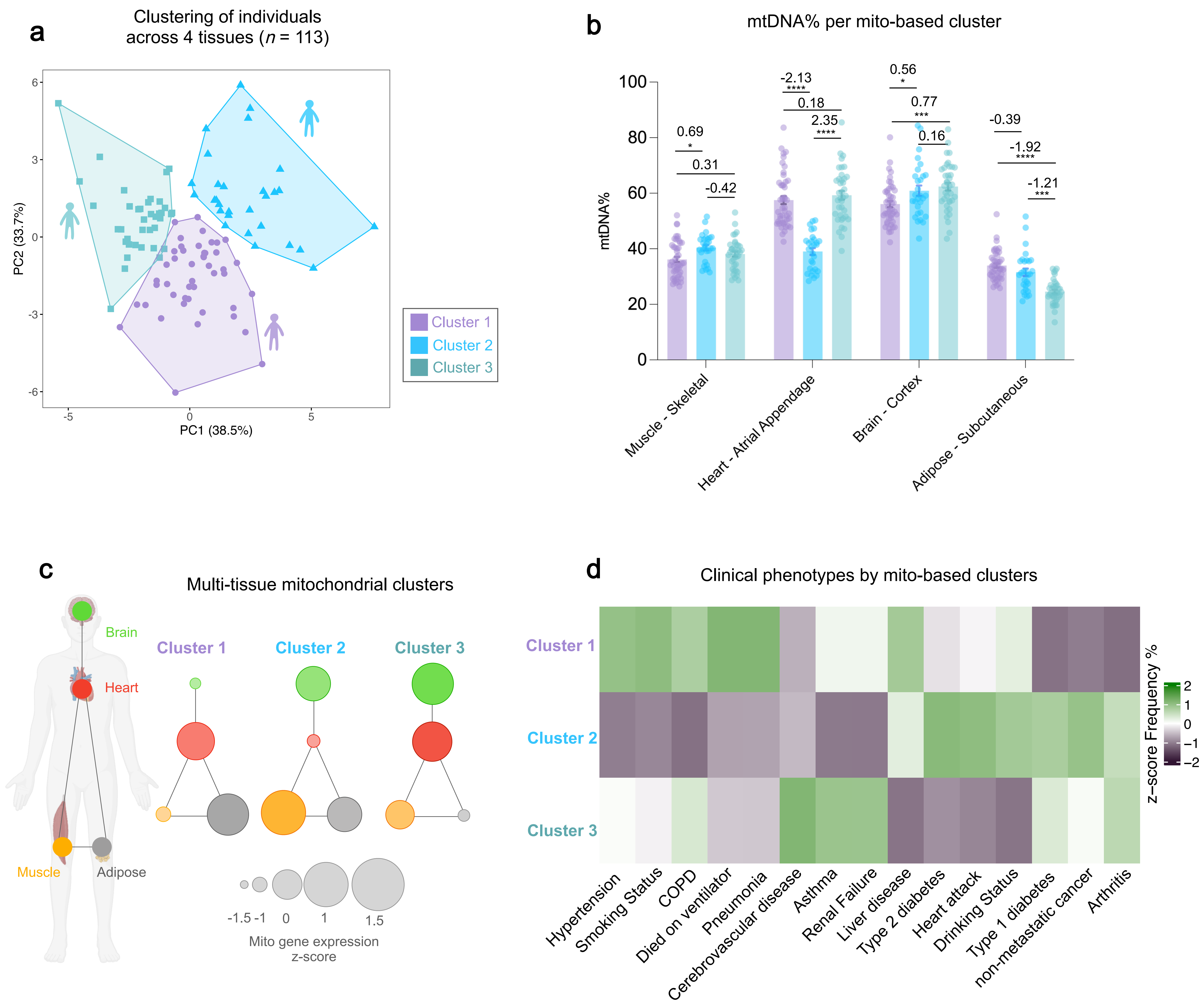

Supplementary Materials

Supplementary File S1 Excel file containing the two mouse mitochondrial enzymatic activity and mtDNA density datasets used in analyses. Sheet 1 contains mouse Cohort 1 dataset. Sheet 2 contains mouse Cohort 2 dataset.

Supplementary File S2 Excel file containing the average, standard deviation, minimum and maximum values of mito-nDNA% (sheet 1) and mtDNA% (sheet 2) in each of the 45 tissues analyzed using the GTEx RNAseq v8 dataset.

Supplementary Figure S1 (a) Correlation matrix of enzymatic activity measures (CI, CII, CIV, and CS) and mtDNA density across five tissues (hippocampus, brown fat, liver, muscle, and bone) from 16 male mice in Cohort 1. (b) Frequency distribution of Spearman *r* correlation coefficients in which only the inter-tissue correlations of the same measure are included (*n* = 50 pairwise tissue comparisons). (c−e) Bivariate plots of mitochondrial enzyme activity measures between tissues**.**

Supplementary Figure S2 Correlation of average mtDNA% with average mito-nDNA% across 45 tissues.

Supplementary Figure S3 Heatmap showing sample size of every pairwise tissue comparison in the same order as Figure 4b.

Supplementary Figure S4 Multi-tissue mitochondrial correlation patterns of mitochondrial pathways. Correlation matrix and frequency distribution of correlation coefficients of mtDNA genes, OxPhos genes, fission and fusion genes, ROS, and glutathione metabolism.

Supplementary Figure S5 Multi-tissue network architecture of mitochondrial gene expression based on mtDNA-encoded genes. Network representation of multi-tissue correlation of mtDNA%. Each node represents a tissue, the size of the node is proportional to its degree, and the edge thickness is proportional to the strength of the correlation.

Supplementary Figure S6 The mtDNAcn-based inter-tissue correlation structure. (a) Heatmap of correlation matrix showing the pairwise Spearman *r* correlations of mtDNAcn between 45 tissues. Grey cells in the heatmap indicate missing values. (b) Frequency distribution of Spearman *r* correlation coefficients between brain-brain (red), body-body (green), and brain-body (yellow) tissues. (c) Network architecture of mtDNAcn-based inter-tissue correlations. (d−f) Bivariate plots of mtDNAcn between tissues.

Supplementary Figure S7 Proteomics-based mitochondrial correlation patterns across seven tissues from 14 GTEx subjects. (a) Heatmap of correlation matrix showing pairwise tissue comparisons of mitochondrial protein abundance. Grey cells in the heatmap indicate missing values. (b) Frequency distribution of Spearman *r* correlation coefficients of inter-tissue correlations of mitochondrial protein abundance. (c−e) Bivariate plots of mitochondrial protein abundance between tissues.

Supplementary Figure S8 Mitochondrial gene expression is driven in part by canonical energy and stress-sensing metabolic pathways. (a) Ranked Spearman *r* correlation coefficients of PGC1a% versus mtDNA% transcripts in each tissue. (b) Biplot showing the correlation of mtDNA% with PGC-1α expression in the colon (transverse). (c) Biplot showing the correlation of mtDNA% with PGC-1α expression in the anterior cingulate cortex. (D) Ranked Spearman *r* correlation coefficients of ISR% versus mtDNA% transcripts in each tissue. (E) Biplot showing the correlation of mtDNA% with ISR expression in the heart (left ventricle). (F) Biplot showing the correlation of mtDNA% with ISR expression in the colon (transverse).

Supplementary Figure S9 Correlation of mitochondrial transcript abundance with regulators of mitochondrial biogenesis and mtDNA maintenance. (a−d) Ranked Spearman *r* between mito-nDNA% (left) or mtDNA% (*right)* in 45 GTEx tissues with the expression levels of (a) *TFAM*, (b) *POLG*, (c) *NRF1*, and (d) *NRF2*. Note that as for PGC1a and ISR genes, in some tissues, the correlations are positive as expected from the literature in the skeletal muscle, heart, and other selected tissues. However, in many tissues, the canonical regulators of mitochondrial biogenesis are not or even negatively correlated with our indices of mitochondrial abundance.

Supplementary Figure S10 Sub-groups of individuals display different mitochondrial distribution patterns. (a) The k-means clustering on mtDNA% ratios between four tissues (muscle - skeletal, heart - atrial appendage, brain - cortex, adipose - subcutaneous tissue) from 113 subjects. Cluster 1, $n = 46$; Cluster 2, $n = 29$; Cluster 3, $n = 38$. (b) Bar plot of mean mtDNA% in each cluster across four tissues. Cluster means of each tissue were tested for significant difference by two-way ANOVA. Effect sizes were computed by Hedge's g. (c) Network visualization of z-score transformed mtDNA% of each cluster across the four tissues analyzed. (d) Heatmap showing the z-score percentage of subjects in each cluster who are recorded as positive for each clinical variable.
